# Supplementary material for: Research on blockchain smart contract technology based on resistance to quantum computing attacks
Source: PLoS One. 2024 May 23;19(5):e0302325. doi: 10.1371/journal.pone.0302325 (PMC11115269; doi:10.1371/journal.pone.0302325)
Supplement: S1 File — (ZIP) [file pone.0302325.s001.zip › data/ntru2/pqntrusign-nist-submission-rev/supporting document/pqNTRUsign.pdf]

NIST PQ Submission: pqNTRUSign
A modular lattice signature scheme

Cong Chen[2], Jeffrey Hoffstein[1], William Whyte[2], and Zhenfei Zhang[2]

[1] Brown University, Providence RI, USA, jhoff@math.brown.edu
[2] OnBoard Security, Wilmington MA, USA,
{cchen,wwhyte,zzhang}@onboardsecurity.com

1 Cover Sheet

This is an overview document of the NTRU lattice-based cryptosystem for the submission of NIST post-quantum cryptography call for standardization. The submitted cryptosystem consists of :

– **pqNTRUSign**: a modular lattice based digital signature scheme that uses the NTRU lattice with either a uniform [15] or a Gaussian sampler [17].

This documents addresses the following requirements:

– **Specifications**
– **Performance analysis**
– **A statement of the advantages and limitations**
– **Cover sheet**
– **Reference implementation**
– **Security analysis**
– **Statement of IPR**

Submission information:

– **Principal submitter:** Zhenfei Zhang, zzhang@onboardsecurity.com, Onboard Security, 187 Ballardvale St. Suite A202, Wilmington, MA, 01887, U.S.
– **Auxiliary submitters:** Chen Cong, Jeffrey Hoffstein and William Whyte.
– **Inventors of the cryptosystem:** Jeffrey Hoffstein, Jill Pipher, John M. Schanck, Joseph H. Silverman, William Whyte and Zhenfei Zhang.
– **Name of the owner of the cryptosystem:** Onboard Security.
– **Backup point of contact:** William Whyte, wwhyte@onboardsecurity.com, Onboard Security, 187 Ballardvale St. Suite A202, Wilmington, MA, 01887, U.S.

The following academic papers contain cryptographic designs, hardness results, security analysis and parameter derivations related to the submitted cryptosystem.

– *NTRU, A ring-based public key cryptosystem*, 1998.

– *Transcript Secure Signatures Based on Modular Lattices*, 2014.
– *A signature scheme from Learning with Truncation*, 2017.
– *Choosing parameters for NTRUEncrypt*, 2017.

Additional information related to implementations, such as public key/private key encodings, conversions, etc. can be found in the additional supporting document:

– *Efficient Embedded Security Standard (EESS) #1.* Version 3.3, 2017.

Information regarding estimating the strength of NTRU lattices against various attacks can be found in our other submission on NTRU encryption algorithms.

2 Algorithm Specifications

2.1 Notation

We use lower case bold letters for vectors, upper case bold letters for matrices. For a polynomial $f(x) = f_0 + f_1 x + \cdots + f_{n-1}x^{n-1}$, we denote its vector form by $\mathbf{f} := \langle f_0, f_1, \ldots, f_{n-1} \rangle$. We sometimes abuse the notation of vector and polynomial when there is no ambiguity. For a polynomial/vector $\mathbf{f}$, the norms are $\|\mathbf{f}\| := \sqrt{\sum_{i=0}^{n-1} f_i^2}$ and $\|\mathbf{f}\|_\infty := \max(|f_i|)$.

We often use the polynomial rings $\mathcal{R}_q := \mathbb{Z}[x]/F(x)$ with $F(x) = x^n \pm 1$. A cyclic rotated matrix of a polynomial $f(x)$ over the ring $\mathcal{R}_q$ is a matrix $\mathbf{M} = (\mathbf{f}_1, \mathbf{f}_2, \ldots, \mathbf{f}_n)^T$ with $\mathbf{f}_i = f(x)x^{i-1} \bmod F(x)$. If $F(x) = x^n - 1$ it is literally cyclic, and it is close to cyclic, up to signs, if $F(x) = x^n + 1$.

For a real a, we let $\lfloor a \rceil$ denote the closet integer to a. For an integer a, we use $[a]_q$ to denote $a \bmod q$; $\lfloor a \rfloor_p := (a - [a]_p)/p$ for the operation of rounding a to the closest multiple of p. Modular operations are center lifted, for example $a \bmod q$ returns an integer within $-q/2$ and $q/2$. These notations are also extended to vectors and matrices.

We use χ_σ^k to denote a k dimensional discrete Gaussian distribution with a deviation of σ. A k-dimensional uniform distribution modulo q is denoted by $\mathcal{U}_q^k$.

2.2 High level Construction

The modular lattice signature scheme was first presented in [15]. Given a lattice $\mathcal{L}$ with a trapdoor $\mathbf{T}$ in the form of a short basis of row vectors; and a message digest in the form of a vector $\mathbf{m}$ whose coefficients are in $[0, p)$, the signature of a modular signature scheme in [15] is a lattice vector $\mathbf{v}$ such that

1. $\mathbf{v} \equiv \mathbf{m} \bmod p$; and
2. $\mathbf{v} \in \mathcal{L}$.

This vector can be obtained via the following steps:

1. sample a vector $\mathbf{v}_0$ from $\mathcal{L}$;
2. use $\mathbf{T}$ to micro-adjust the $\mathbf{v}_0$ so that $\mathbf{v} := \mathbf{v}_0 + \mathbf{aT}$ meets the congruence condition for some $\mathbf{a}$;
3. use rejection sampling to hide $\mathbf{T}$ from $\mathbf{v}$

At a high level, a modular lattice signature scheme is as follows. Let m, n and k be 3 positive integers with $n = k + m$. Let $\mathbf{S}_1 \in \mathbb{Z}_q^{m\times k}$ be a matrix with small (and sparse) coefficients. For simplicity, we assume $\mathbf{S}_1$ is sampled from a certain β-bounded sampler such that $\|\mathbf{S}_1\|_\infty \leq \beta \ll q$. In practice one can use either a discrete Gaussian sampler with small variance, or a uniform sampler within a small range.

Our secret key is a matrix $\mathbf{S} := [p\mathbf{S}_1|\mathbf{I}_m] \in \mathbb{Z}_q^{m\times n}$ with small entries. The public key is constructed from a matrix $\mathbf{A} = \begin{bmatrix}\mathbf{A}_1\\ \mathbf{A}_2\end{bmatrix}$ such that $\mathbf{SA} = 0 \bmod q$ and $\mathbf{A}_2$ is invertible mod q. Equivalently, we can sample $\mathbf{A}_1$ uniformly from $\mathbb{Z}_q^{k\times m}$, and then set $\mathbf{A}_2 = -p\mathbf{S}_1\mathbf{A}_1 \bmod q$. We re-sample $\mathbf{A}_1$ if $\mathbf{A}_2$ is not invertible mod q. The SIS lattice defined by $\mathbf{A}$ is:

$$\mathcal{L} := \{(\mathbf{u}, \mathbf{v}) : \mathbf{uA}_1 + \mathbf{vA}_2 = 0 \bmod q\},$$

where $\mathbf{S}$ is a short trapdoor basis for this lattice. Note that the procedure above is a standard construction for the SIS problem, except that we have a factor of p on $\mathbf{S}_1$. We will show the equivalence between our construction and the standard SIS problem in the next subsection.

It is perhaps more convenient to look at a $k\times m$ matrix $\mathbf{B} := \mathbf{A}_1(-\mathbf{A}_2)^{-1} \bmod q$. With $\mathbf{B}$, the lattice $\mathcal{L}$ can be interpreted as

$$\mathcal{L} := \{(\mathbf{u}, \mathbf{v}) : \mathbf{uB} = \mathbf{v} \bmod q\},$$

with a Learning with Error (LWE) basis

$$\mathbf{P} = \begin{bmatrix} 0 & q\mathbf{I}_m \\ \mathbf{I}_k & \mathbf{B} \end{bmatrix}$$

that allows for efficient sampling.

Signing: We model the hash function H as a random oracle that outputs uniformly over $\mathbb{Z}_p^n$. This allows us to generate random elements $\mathbf{m}_p \in \mathbb{Z}_p^n$ from a message digest μ. We write $\mathbf{m}_p := (\mathbf{u}_p, \mathbf{v}_p)$, with $\mathbf{u}_p \in \mathbb{Z}_p^k$ and $\mathbf{v}_p \in \mathbb{Z}_p^m$.

The next step is to sample a vector $(\mathbf{u}_1, \mathbf{v}_1)$ from $\mathbf{P}$ such $\mathbf{u}_1 \equiv \mathbf{u}_p \bmod p$. To do so, one can simply sample a vector $\mathbf{r}$ from a given distribution (χ_σ^k or $\mathcal{U}_q^k$). Then, compute $\mathbf{u}_0 = p\mathbf{r}$, $\mathbf{u}_1 = \mathbf{u}_0 + \mathbf{u}_p$, and then find a lattice vector whose "s-side" is $\mathbf{u}_1$ by setting $\mathbf{v}_1 = \mathbf{u}_1\mathbf{B} \bmod q$. As such, $(\mathbf{u}_1, \mathbf{v}_1)$ is a vector in the lattice, with $\mathbf{u}_1 \equiv \mathbf{u}_p \bmod p$.

We expect $\mathbf{v}_1$ to have random coefficients modulo p, and therefore it will not meet the congruence condition. To complete the process, we need to micro-adjust $\mathbf{v}_1$ so that the t-side also meets the congruence condition; in the meantime we

do not want to break the congruence condition on the s-side. We use the secret basis $\mathbf{S} = [p\mathbf{S}_1|\mathbf{I}_m]$ to achieve this goal. Let $\mathbf{a} = \mathbf{v}_p - \mathbf{v}_1 \bmod p$. We compute $(\mathbf{u}_2, \mathbf{v}_2) = \mathbf{aS} = (p\mathbf{aS}_1, \mathbf{a})$. Note that $(\mathbf{u}_2, \mathbf{v}_2) \equiv (0, \mathbf{a}) \bmod p$ by construction, and $(\mathbf{u}_2, \mathbf{v}_2)$ is a vector in the lattice.

The final signature is $(\mathbf{u}, \mathbf{v}) = (\mathbf{u}_1, \mathbf{v}_1) + (\mathbf{u}_2, \mathbf{v}_2)$. It is easy to see that $(\mathbf{u}, \mathbf{v})$ remains in the lattice as long as $\|\mathbf{v}\|_\infty < q/2$. On the other hand, we have

$$\mathbf{u} = \mathbf{u}_1 + \mathbf{u}_2 = \mathbf{u}_1 \equiv \mathbf{u}_p \bmod p$$

and

$$\mathbf{v} = \mathbf{v}_1 + \mathbf{v}_2 \equiv \mathbf{v}_1 + \mathbf{v}_p - \mathbf{v}_1 \equiv \mathbf{v}_p \bmod p.$$

Therefore, $(\mathbf{u}, \mathbf{v})$ is a valid signature for our scheme.

Remark 1. As stated before, a candidate signature $(\mathbf{u}, \mathbf{v})$ leaks information about the secret key $\mathbf{S}$. To seal this leak one needs to use a rejection sampling technique [19]. The efficiency of the above scheme relies heavily on how often one will need to reject a signature. We will give more details on this in the next section.

2.3 Ring variant

In the following we will work over the polynomial ring $\mathcal{R}_q = \mathbb{Z}_q[x]/(x^N + 1)$. Our scheme also works over other rings, such as $\mathbb{Z}_q[x]/(x^N - 1)$ with minor modification. Let $f(x)$, $g(x)$ and $h(x)$ be 3 polynomials in $\mathcal{R}_q$, where $f(x)$ and $g(x)$ have very small coefficients; $h(x) = p^{-1}g(x)f^{-1}(x)$. We express by $\mathbf{f}$, $\mathbf{g}$ and $\mathbf{h}$ the vector form of the polynomials. Also let $\mathbf{F}$, $\mathbf{G}$ and $\mathbf{H}$ be the matrix obtained from nega-cyclic rotations. The NTRU lattice with regard to h is defined as

$$\mathcal{L}_h = \{(u, v) \in \mathcal{R}_q^2 : uh = v\}$$

or rather, the vector/matrix form:

$$\mathcal{L}_h = \{(\mathbf{u}, \mathbf{v}) : \mathbf{uH} = \mathbf{v} \bmod q\}$$

where there exists a public basis $\mathbf{P} = \begin{bmatrix} 0 & q\mathbf{I}_N \\ \mathbf{I}_N & \mathbf{H} \end{bmatrix}$ and a secret generator $[p\mathbf{F}|\mathbf{G}]$. We also require $g(x)$ to be invertible over $\mathcal{R}_p$, which is the same as having $\mathbf{G}$ being invertible mod p.

Now we will use $[p\mathbf{F}|\mathbf{G}]$ (rather than $[p\mathbf{S}_1|\mathbf{I}_m]$) to perform the micro-adjustment. This modification does raise another issue: the "t-side" vector during the signing procedure will contain information about $\mathbf{G}$. To be precise, the "t-side" vector will be $\mathbf{v} := \mathbf{v}_1 \pm \mathbf{ag}$ where $\mathbf{v}_1$ is indistinguishable from uniform over $\mathcal{R}_q$, and $\mathbf{a}$ is uniform over $\mathbb{Z}_p^N$. We will need to perform rejection sampling to seal the leakage of information about $\mathbf{g}$. As shown in [15], after rejection sampling, the distribution of $\mathbf{v}$ will be computationally indistinguishable from uniform over $(-\frac{q}{2} + B_t, \frac{q}{2} - B_t)$ for a public parameter B_t which depends on q, the (uniform) distribution of $\mathbf{a}$, and the number of $+1$s and -1s in $\mathbf{g}$.

To avoid confusion, we will use M_s to denote the rejection rate for the s-side, M_t for the t-side, and M for the overall rate.

2.4 The schemes

Now we are ready to present our proposed algorithms. Our scheme uses two types of parameters, one that employs Gaussian samplers and the other with uniform samplers. Algorithms for those two types of parameter sets differ slightly due to the rejection sampling process. Below, we present first the key generation algorithm, which remains the same for all parameters, and then algorithms for Gaussian samplings and uniform samplings, respectively.

Key generation : The key generation algorithm is shown in Algorithm 1. We use

Algorithm 1 Key Generation Algorithm

Input: Parameters N, p, q, d and B_k
Output: Public key $\mathbf{h}$ and secret key $(p\mathbf{f}, \mathbf{g})$
1: $\mathbf{f} \leftarrow T(d+1, d)$
2: **if** $\mathbf{f}$ is not invertible mod q **then** go to step 1 **end if**
3: **if** NormF($\mathbf{f}$) $\geq B_k$ **then** go to step 1 **end if**
4: $\mathbf{g} \leftarrow T(d+1, d)$
5: **if** $\mathbf{g}$ is not invertible mod p **then** go to step 4 **end if**
6: **if** NormF($\mathbf{g}$) $\geq B_k$ **then** go to step 4 **end if**
7: $\mathbf{h} = \mathbf{g}/(p\mathbf{f}) \bmod q$
8: **return** $\mathbf{h}$, $\mathbf{g}$ and $\mathbf{f}$

the classical NTRU flat form (non-product form, cf. [16]) keys with a pre-fixed number of +1s and −1s. Here, $T(d_1, d_2)$ is a set of trinary polynomials of degree less than N, where there are exactly d_1 positive coefficients and d_2 negative coefficients. One can choose thicker keys for a higher level of security. Since we require both $\mathbf{f}$ and $\mathbf{g}$ to be invertible, we have set $f(1) = g(1) = 1$.

In BLISS [8], there is an extra rejection sampling process on keys $\mathbf{f}$ and $\mathbf{g}$ during key generation, so that $\|\mathbf{af}\|$ is reasonably bounded for efficient rejection sampling on signatures. We use a similar approach, namely NormF for rejections. NormF is computed as $\|\mathbf{t}\|_\infty$ where $\mathbf{t} = \sum_{i=0}^{N-1} x^i f(x) \bmod (x^N + 1)$.

We view $\mathbf{af}$ as a vector-matrix multiplication with vector $\mathbf{a}$ and matrix $\mathbf{F}$, the anti-cyclic rotation of $\mathbf{f}$. Since $\mathbf{a}$ is binary, this multiplication can be seen as randomly adding some coefficients for each column of $\mathbf{F}$. Therefore, for a given $\mathbf{f}$, the corresponding $\mathbf{t}(x)$, where the i-th coefficient of $\mathbf{t}(x)$ is the sum of the i-th column of $\mathbf{F}$. will be correlated to the expected value of $\|\mathbf{af}\|_\infty$. Rejection sampling on secret keys based on the maximum norm of $\mathbf{t}(x)$ will ensure that $\|\mathbf{af}\|_\infty$ is also likely to be small.

The scheme with Gaussian sampling

Signing algorithm : We make the following remarks about this signing algorithm:

First, there is a factor of $\mathbf{g}^{-1} \bmod p$ for step 4, which is there to ensure the congruence condition for the t-side.

Algorithm 2 Signing for parameters with Gaussian distributions

Input: Message μ; Public key $\mathbf{h}$; Secret key $\mathbf{f}$ and $\mathbf{g}$; Distribution χ_σ
Input: Parameters N, p, q, M_s, B_s, B_t
Output: A signature $\mathbf{b}$ for message μ
1: $(\mathbf{u}_p, \mathbf{v}_p) = \text{Hash}(\mu|\mathbf{h})$
2: $\mathbf{r} \leftarrow \chi_\sigma^N$, $b \leftarrow \{0,1\}$
3: $\mathbf{u}_1 = p\mathbf{r} + \mathbf{u}_p$; $\mathbf{v}_1 = \mathbf{u}_1\mathbf{h} \bmod q$
4: $\mathbf{a} = (\mathbf{v}_p - \mathbf{v}_1)/\mathbf{g} \bmod p$
5: **if** $\|\mathbf{af}\|_2 > B_s$ or $\|\mathbf{ag}\|_\infty > B_t$ **then** go to step 2 **end if**
6: $\mathbf{v} = \mathbf{v}_1 + (-1)^b\mathbf{ag}$;
7: **if** $\|\mathbf{v}\|_\infty > q/2 - B_t$ **then** go to step 2 **end if**
8: **return** $\mathbf{b} = (\mathbf{r} + (-1)^b\mathbf{af})$ with probability $1/\left(M_s \exp\left(-\frac{\|\mathbf{af}\|}{2\sigma^2}\right)\cosh\left(\frac{\langle \mathbf{b},\mathbf{af}\rangle}{\sigma^2}\right)\right)$
9: go to step 2

Second, in step 5, we check the norm requirements for $\mathbf{af}$ and $\mathbf{ag}$. This is to ensure that the rejection samplings in the followed steps deliver the desired acceptance rate. Note that B_s is a public parameter depending only on d_1, d_2. It is determined experimentally by an average over a large number of samples of $\mathbf{f}$ chosen randomly from $T(d_1, d_2)$ and $\mathbf{a}$ chosen randomly and uniformly from $\mathbb{Z}_p^N$.

Third, in step 7, rejection sampling is performed on the t-side, parameterized by an additional integer B_t. The distribution of the t-side vector will be uniform within the interval $(-\frac{q}{2} + B_t, \frac{q}{2} - B_t)$. The public parameter B_t is also computed as an average over a large number of choices of $\mathbf{g}$ and $\mathbf{a}$, as described in Section 6.1.

Finally, we have

$$(\mathbf{u}, \mathbf{v}) = (\mathbf{u}_1, \mathbf{v}_1) + (-1)^b(\mathbf{u}_2, \mathbf{v}_2)$$

for a random bit b. This makes the raw distribution of $\mathbf{b} := (\mathbf{r} + (-1)^b\mathbf{af})$ a bimodal Gaussian distribution. This allows us to achieve a higher acceptance rate for this distribution. Note that in the initial construction of BLISS [8], the bimodal Gaussian distribution makes a signature sometimes unverifiable due to the odd modulus q. BLISS solved this problem by moving the modulus from q to $2q$. We solve this problem by setting $p = 2$. It follows that $\mathbf{v} \equiv \mathbf{v}_1 + (-1)^b(\mathbf{v}_p - \mathbf{v}_1) \equiv \mathbf{v}_p \bmod 2$.

The scheme with Uniform sampling This version of the scheme uses the same key generation process as the one described in the previous section.

Algorithm 3 Verification for parameters with Gaussian distributions

Input: Message μ; Public key $\mathbf{h}$; Signature $\mathbf{b}$; Parameters p, q, B_t, σ, N
Output: Accept or Reject the signature
1: $(\mathbf{u}_p, \mathbf{v}_p) = \text{Hash}(\mu|\mathbf{h})$
2: $\mathbf{u} = p\mathbf{b} + \mathbf{u}_p$
3: **if** $\|\mathbf{u}\|^2 > p^2\sigma^2 N$ **then** Reject **end if**
4: $\mathbf{v} = \mathbf{uh} \bmod q$
5: **if** $\mathbf{v} \not\equiv \mathbf{v}_p \bmod p$ or $\|\mathbf{v}\|_\infty > q/2 - B_t$ **then** Reject **end if**
6: **return** Accept

Algorithm 4 Signing for parameters with uniform distributions

Input: $(\mathbf{f}, \mathbf{g}, \mathbf{h}, \mu)$, where $(\mathbf{f}, \mathbf{g})$ is a private key, $\mathbf{h}$ is the corresponding public key, and $\mu \in \{0,1\}^*$ is a document to be signed.
1: $(\mathbf{u}_p, \mathbf{v}_p) \longleftarrow \text{Hash}\big(\mathbf{h}, \mu\big)$
2: **repeat**
3: $\quad \mathbf{r} \xleftarrow{\$} \mathcal{U}^N_{\lfloor q/(2p)+0.5 \rfloor}$
4: $\quad \mathbf{u}_0 \longleftarrow \mathbf{u}_p + p\mathbf{r}$
5: $\quad \mathbf{v}_0 \longleftarrow \mathbf{hu}_0 \pmod q$
6: $\quad \mathbf{a} \longleftarrow \mathbf{g}^{-1}(\mathbf{v}_p - \mathbf{v}_0) \pmod p$
7: $\quad (\mathbf{u}, \mathbf{v}) \longleftarrow (\mathbf{u}_0, \mathbf{v}_0) + (p\mathbf{af}, \mathbf{ag})$
8: **until** $\big\|p\mathbf{af}\big\| \le B_s$ and $\big\|\mathbf{ag}\big\| \le B_t$ and $\big\|\mathbf{u}\big\| \le \dfrac{q}{2} - B_s$ and $\big\|\mathbf{v}\big\| \le \dfrac{q}{2} - B_t$
Output: $(\mathbf{u}, \mathbf{v}, \mu)$

Remark 2. Notice the rejection criterion in Step 8 of the signing algorithm. We compute a potential signature $(\mathbf{u}, \mathbf{v})$, but then we reject it if it, or the correction $(\mathbf{af}, \mathbf{ag})$, is too big; specifically, we reject $(\mathbf{u}, \mathbf{v})$ if it falls outside of $L_{\mathbf{h}}\left(\frac{q}{2} - B_s, \frac{q}{2} - B_t\right)$, or if $(\mathbf{af}, \mathbf{ag})$ falls outside $L_{\mathbf{h}}\left(B_s, B_t\right)$.

Algorithm 5 Verification for parameters with uniform distributions

Input: $(\mathbf{u}, \mathbf{v}, \mu, \mathbf{h})$
1: *valid* $\longleftarrow$ yes
2: $(\mathbf{u}_p, \mathbf{v}_p) \longleftarrow \text{Hash}(\mathbf{h}, \mu)$
3: **if** $\mathbf{v} \not\equiv \mathbf{hu} \pmod q$ **then**
4: $\quad$ *valid* $\longleftarrow$ no
5: **end if**
6: **if** $\|\mathbf{u}\| > \dfrac{q}{2} - B_s$ or $\|\mathbf{v}\| > \dfrac{q}{2} - B_t$ **then**
7: $\quad$ *valid* $\longleftarrow$ no
8: **end if**
9: **if** $(\mathbf{u}, \mathbf{v}) \not\equiv (\mathbf{u}_p, \mathbf{v}_p) \pmod p$ **then**
10: $\quad$ *valid* $\longleftarrow$ no
11: **end if**
Output: *valid*

3 Design Rationale

3.1 Hardness assumption

Overview We first give an overview of the hardness assumptions in this proposal.

- The hardness of a modular lattice signature scheme over a generic lattice can be reduced to the short integer solution problem over this lattice. This is not used in this submission, as it is too inefficient; however it gives confidence in the overall design of the framework.
  - The public key security is based on the short integer solution problem.
  - The forgery security is based on the learning with truncation problem.
  - The transcript security is provided by rejection samplings.
- pqNTRUSign is an efficient instantiation of modular lattice signature over the NTRU lattice. This is the submission.
  - The public key security is based on the NTRU assumption.
  - The forgery security is based on the learning with truncation problem over NTRU lattice.
  - The transcript security is provided by rejection samplings.

Details For the sake of completeness, we present the related foundational hard problems. A lattice $\mathcal{L}$ is a discrete sub-group of $\mathbb{R}^n$, or equivalently, the set of all the integral combinations of $d \leq n$ linearly independent vectors over $\mathbb{R}$:

$$\mathcal{L} := \mathbb{Z}\mathbf{b}_1 + \mathbb{Z}\mathbf{b}_2 + \cdots + \mathbb{Z}\mathbf{b}_d, \mathbf{b}_i \in \mathbb{R}^n.$$

$\mathbf{B} := (\mathbf{b}_1, \ldots, \mathbf{b}_d)^T$ is called a basis of $\mathcal{L}$.

Definition 1 (γ-SVP and uSVP). *Given a lattice $\mathcal{L}$, finding a vector that is no longer than $\gamma \cdot \lambda_1(\mathcal{L})$ is called the* approximate shortest vector problem (γ-SVP)*, where λ_1 is the first minima, i.e, the length of the shortest vector, of the lattice.*

Given a particular lattice $\mathcal{L}$, where there exists a unique shortest non-zero vector, finding this vector is called the unique shortest vector problem.

We view an NTRU lattice as an $\mathcal{R}_q$ module of rank 2. Let $\mathbf{f}, \mathbf{g} \in \mathcal{R}_q$ with small coefficients. Let $\mathbf{h} = \mathbf{g}/\mathbf{f}$ over $\mathcal{R}_q$. The NTRU lattice associated with $\mathbf{h}$ is defined as

$$\mathcal{L} := \{(\mathbf{s}, \mathbf{t}) \in \mathcal{R}_q^2 : \mathbf{t} \equiv \mathbf{s}\mathbf{h} \mod q\}.$$

Definition 2 (NTRU assumption). *Given $\mathbf{h}$, it is believed to be hard to find $\mathbf{f}$ and $\mathbf{g}$.*

The NTRU assumption can be reduced to the uSVP for the NTRU lattice.

Definition 3 (LWT$_{q,p,n,m}$). *Let q, p, n, m be positive integers with p co-prime to q. Sample uniformly at random a matrix $\mathbf{A} \in \mathbb{Z}_q^{n\times m}$ and a vector $\mathbf{s} \in \mathbb{Z}_q^n$; compute $\mathbf{b} = \mathbf{sA} \bmod q \bmod p$; the decisional LWT problem is: given two pairs $(\mathbf{A}, \mathbf{b})$ and $(\mathbf{A}, [\mathbf{u}]_p)$, where $\mathbf{u}$ is sampled uniformly at random in $\mathbb{Z}_q^n$, distinguish those two pairs. The computational problem is: given $(\mathbf{A}, \mathbf{b})$, find $\mathbf{s}$.*

It was shown in [17] that the LWT problem is equivalent to the LWR problem with $r \equiv p^{-1} \mod q$, where LWR is defined as follows:

Definition 4 (LWR$_{q,r,n,m}$). *Sample uniformly at random a matrix $\mathbf{A} \in \mathbb{Z}_q^{n\times m}$ and a vector $\mathbf{s} \in \mathbb{Z}_q^n$; compute $\mathbf{b} = \lfloor \mathbf{sA} \bmod q \rfloor_r$; the decisional LWR problem is: given two pairs $(\mathbf{A}, \mathbf{b})$ and $(\mathbf{A}, \lfloor \mathbf{u} \rfloor_r)$ where $\mathbf{u}$ is sampled uniformly at random in $\mathbb{Z}_q^n$, distinguish those two pairs. The computational problem is: given $(\mathbf{A}, \mathbf{b})$, find $\mathbf{s}$.*

It has been shown in [3] that the decisional LWR$_{q,r,n,m}$ problem is hard assuming the hardness of LWE$_{q,r,n,m'}$ with parameters

$$m \geq \frac{\log(q)}{\log(2\gamma)} \cdot m' \qquad \text{and} \qquad q \geq \gamma(nm\beta p)$$

for some $\gamma \geq 1$.

3.2 Rejection sampling and sampler agility

Early lattice based signature schemes, such as GGHSign [12] and NTRUSign [14], leak private key information in a transcript of message/signature pairs. An attacker can produce a signing key from a long enough transcript using methods for "learning a parallelepiped" [22,10].

In [20], Lyubashevsky proposed a rejection sampling method to thwart transcript leakage attacks. Using his technique, signatures are produced according to a fixed public distribution (typically either a Gaussian as in [20] or a uniform distribution as in [15]). A transcript reveals only this public distribution, and contains no information about the particular signing key that is used to generate the signatures.

The *sampling method* therefore becomes a core issue in designing signature schemes. For example, replacing a Gaussian sampler with a bimodal Gaussian sampler [8] significantly improves the performance of a scheme. However, although schemes using Gaussian sampling allow smaller signature sizes, recent developments in lattice based signature schemes [9] shows a trend toward moving back to uniform rejection sampling, since uniform sampling is easier to implement and to ensure constant time. In comparison, there are side-channel attacks on some lattice based signature schemes exploiting the Gaussian look-up tables [4].

In this submission, we build in *sampler agility* by allowing the user to choose either a (bimodal) Gaussian sampler or a uniform sampler. In particular, we provide a structured API for Gaussian samplers with the hope that more efficient and secure samplers are developed during the process of this call for proposals

from NIST. We also remark that in the use cases where side channel attacks are not a concern, one can still use a (bimodal) Gaussian sampler for better performance.

3.3 Transcript simulation

By Theorem 4.6 of [21] a transcript produced by this signing algorithm will consist of a triple $(\mathbf{u}_p, \mathbf{v}_p, \mathbf{b})$, with distribution $U_p^N \times U_p^N \times \mathcal{D}^N$, where U_p is uniform mod p and $\mathcal{D}^N$ is our input distribution, i.e., a discrete Gaussian distribution or uniform distribution. Such a transcript, indistinguishable from a genuine transcript, can be simulated without knowledge of the secret key in the following way:

1. Choose $\mathbf{b}$ at random from $\mathcal{D}^N$
2. Set $\mathbf{u} = p\mathbf{b} + \mathbf{u}_p$, with entries of $\mathbf{u}_p$ chosen at random mod p
3. Set $\mathbf{v} \equiv \mathbf{uh} \mod q$, and lift $\mathbf{v}$ to the interval $(-q/2, q/2]$
4. Reject if $\|\mathbf{v}\|_\infty > q/2 - B_t$ and return to Step 1. Otherwise set $\mathbf{v}_p \equiv \mathbf{v} \mod p$ and accept $\mathbf{b}$ as a signature on $\mathbf{u}_p, \mathbf{v}_p$

It is easy to see that such a simulation will generate a triple $(\mathbf{u}_p, \mathbf{v}_p, \mathbf{b})$ that appears to be a legitimate transcript, in the case of uniform distribution.

In the case of bimodal Gaussian distribution, because of the potential for rejection of a genuine signature in steps 2 and 3 of the verification algorithm, it appears at first that some message digests $(\mathbf{u}_p, \mathbf{v}_p)$, originally chosen by the hash function to be uniform from $U_p^n \times U_p^n$, might be rejected, (by the L^2 condition), leading to an unknown asymmetrical distribution of the $(\mathbf{u}_p, \mathbf{v}_p)$ in genuine signatures. However, the parameters are selected so that the chances of this occurring are less than 2^{-128}. Therefore to simulate transcripts computationally indistinguishable from genuine ones, it suffices to select, in the simulation process, $\mathbf{u}_p$ uniformly from U_p^n. In step 4 of the simulation process, the $\mathbf{v}_p$ is chosen by setting $\mathbf{v}_p \equiv \mathbf{uh} \mod q \mod p$, and via rejection sampling, $\mathbf{uh} \mod q$ is guaranteed to have entries uniformly and randomly distributed in the interval $[-q/2 + B_t, q/2 - B_t]$. We are making the assumption, born out by experiment, that after reduction mod p this produces a $\mathbf{v}_p$ with entries uniformly distributed mod p.

3.4 Parameters

We give 2 sets of parameters, namely, Gaussian-1024 and Uniform-1024 in Table 1. We estimate that

- Gaussian-1024 and Uniform-1024 provide 269 bits of classical security and 149 bits of quantum security;

The details of the above estimations shall be presented in the next subsection. Note that the parameter sets in this proposal are not the same as in [15,17] due to the use of a different estimation model. In previous estimations we simply

followed the work of [5] and [8] by assuming a root Hermite factor of 1.005 is not achievable. Here we use a more rigorous estimation for our new parameter sets.

Table 1. Parameters.

Param	$\mathcal{R}, N, q$	d_f, d_g	σ	B_k, B_s, B_t	Raw PK size	Raw Sig size
Gaussian-1024	$\frac{\mathbb{Z}_q[x]}{x^N+1}, 1024, 2^{16}+1$	205	250	40, 500,49	16384 bits	$\approx$ 11264 bits
Uniform-1024	$\frac{\mathbb{Z}_q[x]}{x^N+1}, 1024, 2^{16}+1$	205	N/A	40, 98, 49	16384 bits	16384 bits

Table 2. MACRO definitions for NIST's API

Param	Gaussian-1024	Uniform-1024
CRYPTO_SECRETKEYBYTES	2604	2604
CRYPTO_PUBLICKEYBYTES	2065	2065
CRYPTO_BYTES	2065	2065

We list the related Marcos in Table 2. Note that the secret key field contains both public key **h** and private keys **f** and **g**, as well as intermediate data $\mathbf{g}^{-1}$ mod p in the secret key. For minimum storage, one can simply store **f** and **g**, and compute **h** and $\mathbf{g}^{-1}$ on the fly. Also note that the signature size for Gaussian-1024 is greater than that in Table 1 because we did not implement Hoffman encoding.

For all 5 NIST's required security levels, we suggest the use of Gaussian-1024 or Uniform-1024 parameter sets.

3.5 Best known attacks

Overview In this evaluation, we will

1. follow the original BKZ 2.0 analysis [5] with the extreme pruning method to estimate the **classical security**;
2. follow the new analysis in [2] using BKZ 2.0 with quantum sieving to estimate the **quantum security**.

For completeness, we also give the analysis result of

3. the new analysis in [2] using BKZ 2.0 with classical sieving.

However, we will **not** use this result to estimate the classical security, due to the excessive space requirement. For more details and justifications please see our other submission on NTRU encryption algorithms.

For signatures, there are two types of attacks, namely, public key attacks which try to recover the secret key from a public key, and forgery attacks, which

Table 3. Lattice strength given by root Hermite factor

	Gaussian-1024	Uniform-1024
Public key strength $\left(\frac{\mathrm{GH}}{\lambda_1}\right)^{\frac{1}{2N}}$	$524^{\frac{1}{2048}} \approx 1.0030$	
Forgery strength $\left(\frac{\|\mathbf{u},\mathbf{v}\|}{\mathrm{GH}}\right)^{\frac{1}{2N}}$	$25^{\frac{1}{2048}} \approx 1.0016$	$153^{\frac{1}{2048}} \approx 1.0024$

try to forge a signature without a secret key. As we shall see from Table 3, forgery attacks are strictly harder than public key attacks, due to the smaller root Hermite factors. Therefore, our focus here is to evaluate the security of the public keys, i.e., recovering $\mathbf{f}$ or $\mathbf{g}$ from $\mathbf{h}$.

Here, we summarize the evaluations in Table 4. For more details on the cost analysis of BKZ and hybrid attacks, please see our other submission on NTRU encryption algorithms. For completeness, we also present the best known forgery attack.

Table 4. Best known attacks and their costs on public keys

N	BKZ + Enum		BKZ + Sieving		BKZ + QSieving	
	uSVP	Hybrid	uSVP	Hybrid	uSVP	Hybrid
1024	407	**269**	165	165	**149**	154

Public keys. The best known attacks against NTRU lattices are the hybrid attack [18] combining lattice reductions and meet-in-the-middle attacks, and lattice reduction attacks using BKZ with (quantum) sieving. The strength of these attacks, as well as the analysis of parameters for NTRU lattices that are secure from these attacks, are shown in [16] and our other submission on NTRU encryption algorithms.

Essentially, the public key strength is determined by the hardness of the unique shortest vector problem of the NTRU lattice, which is related to the $2N$-th root of the following quantity:

$$\frac{\text{Gaussian Heuristic Length}}{\lambda_1} = \frac{\sqrt{2N/(2\pi e)}q^{N/(2N)}}{\|\mathbf{f},\mathbf{g}\|_2} = \sqrt{\frac{Nq/(\pi e)}{2d_f + 2d_g + 2}}.$$

Forgeries. The best forgery attack, other than deriving the secret keys from the public keys via the above attacks, is the lattice reduction attack shown in [15]. Forging a signature can be accomplished if an associated approximate closest vector problem in the intersection of the NTRU lattice, and $p\mathbb{Z}^{2N}$ can be solved. Therefore, the task of forgery can be solved by finding a vector that meets the congruence mod p requirements, and is sufficiently close to the intersection lattice to satisfy the length requirement.

This problem is harder than that of finding a short vector in the intersection lattice, and so to simplify our analysis we will use this to quantify the strength of the lattice problem. The intersection lattice is generated by the rows of the matrix

$$\begin{bmatrix} 0 & pq\mathbf{I}_N \\ p\mathbf{I}_N & p\mathbf{H}' \end{bmatrix},$$

for some appropriate $\mathbf{H}'$. We also assume that this lattice behaves like a random lattice.

For Uniform-1024, each coordinate of $\mathbf{u}$ (and $\mathbf{v}$) is approximately randomly and uniformly distributed between $-q/2+B_s$ and $q/2-B_s$ ($-q/2+B_t$ and $q/2-B_t$, resp.) . Ignoring the B_s, the average squared coefficient will be approximately

$$\frac{1}{q}\int_{-q/2}^{q/2} x^2 dx = q^2/12.$$

Thus $\mathbf{u}$ and $\mathbf{v}$ will have norm $\|\mathbf{u}\|^2 \approx \|\mathbf{v}\|^2 \approx q^2N/12$. We thus have

$$\frac{\text{Target Length}}{\text{Gaussian Heuristic Length}} = \frac{\sqrt{q^2N/6}}{\sqrt{Np^2q/\pi e}} = \sqrt{\frac{q\pi e}{6p^2}}.$$

For Gaussian-1024, notice that the lattice is not "balanced" as $\|\mathbf{u}\|$ is significantly smaller than $\|\mathbf{v}\|$. In general, if the target is a vector $(\mathbf{u},\mathbf{v})$, with $\mathbf{u},\mathbf{v}$ each N-dimensional, and satisfying $\|\mathbf{u}\| \approx a\sqrt{N}$ and $\|\mathbf{v}\| \approx b\sqrt{N}$, then the optimal matrix for maximizing strength against lattice reduction attacks, that is, minimizing the ratio of the norm of the target of the Gaussian expected norm, is the $2N$ by $2N$ matrix

$$\begin{bmatrix} 0 & pq\mathbf{I}_N \\ \alpha p\mathbf{I}_N & p\mathbf{H} \end{bmatrix},$$

with α chosen so that $\alpha = b/a$.

The vector $(\alpha\mathbf{u},\mathbf{v})$ will be a short vector in this matrix, and it is not surprising that the optimal α equalizes the lengths of the vectors $\alpha\mathbf{u}$, and $\mathbf{v}$. We omit the details justifying this.

We now determine the values of a, b in our case. As it is a vector with each coordinate choses from the Gaussian distribution, the expected norm of $\|\mathbf{u}\|$ will satisfy $\|\mathbf{u}\|^2 \approx p^2\sigma^2N$. Thus $a = p\sigma$. Also, $\mathbf{v}$ will have norm $\|\mathbf{v}\|^2 \approx q^2N/12$, so $b = q/\sqrt{12}$.

As stated above, in our particular case $a = p\sigma$, $b = q/\sqrt{12}$, so $\alpha = q/(p\sigma\sqrt{12})$, and the length of the target is

$$\text{Length target} \approx b\sqrt{2N} = q\sqrt{N/6}.$$

For general, a, b, and $\alpha = b/a$, the determinant of the matrix is $\alpha^N p^{2N} q^N$, and thus the length of the Gaussian expected shortest vector is

$$\text{Gaussian Heuristic Length} = \alpha^{1/2}pq^{1/2}\sqrt{\frac{2N}{2\pi e}} = \sqrt{\frac{Npq^2}{\pi e\sigma\sqrt{12}}}$$

We thus have

$$\frac{\text{Target Length}}{\text{Gaussian Heuristic Length}} = \sqrt{\frac{\pi e \sigma}{p\sqrt{3}}},$$

and the strength against forgery is determined by the $2N^{th}$ root of this ratio, which equals

$$\left(\frac{\pi e \sigma}{p\sqrt{3}}\right)^{1/(4N)}.$$

We estimate that our parameter set delivers 128 bits security against classical and quantum attackers, assuming the complexity of BKZ 2.0 using enumeration with extreme pruning [5,11]. This is using the same metric as was used in [8] and [15].

3.6 Advantages and limitations

Smaller signature size by design. Recall that in [15], a signature in this scheme is a lattice vector. Since the verifier already knows a (bad) basis of the lattice for verification purpose, it is sufficient to transmit part of the vector $\mathbf{v}$ as long as the verifier can complete the whole vector during the verification phase.

Popular lattice based schemes, such as BLISS [8] and TESLA [1], do not have this property. Signatures in those schemes are vectors *close* to the lattice. Hence, when the vectors are compressed, an additional helper needs to be generated for the verifier to derive the original vector (although this helper is only a few hundred bits). To be precise, if we parameterize the scheme to be presented in this paper with the same parameters as in [8], the difference in the size of a signature is exactly the size of this helper.

Hash-then-sign methodology. In the area of classical cryptography, there are two main methods to build digital signatures, namely, Schnorr signatures and hash-then-sign signatures. However, in terms of lattice based signatures, almost all popular candidate use the framework of the Shnorr signature. The pqNTRUSign scheme we submit gives an alternative solution to the Schnorr based solutions.

NTRU *trapdoor.* In general, lattice based signatures offer the best performance among quantum-safe solutions, in terms of the combination of signature sizes and public key sizes. However, the performance changes greatly with how the trapdoor is constructed. The NTRU trapdoor is in general the most efficient one in the literature; It has survived 20 years of cryptanalysis, which no other lattice-based solution has gone through.

Sampler agility. Our proposal provides sampler agility. We provide solutions for both uniform samplers and Gaussian samplers; and an easy migration path should new and more efficient Gaussian samplers are invented. This allows for security oriented adoptions (with uniform sampling) and performance oriented adoptions (with Gaussian sampling).

3.7 Performance and implementations

We present the implementation results in Table 5.

Table 5. Benchmark results

	Gaussian-1024	Uniform-1024
Key Generation	47.8 ms	48.9 ms
Signing	120ms	72 ms
Verification	0.96 ms	0.97 ms

Optimizations not in this submission package. There are three optimizations that we are aware of, that are not included in this submission package. Namely

1. AVX2 based optimization for polynomial multiplication [7]; this accelerates polynomial multiplication by 2.3 times.
2. GPU based optimization [6]; this accelerates signature generation by 31 times.
3. Product form polynomials [16]; this speeds polynomial multiplications by around 3 times.
4. Signature aggregation [17]; this allows one to verify a batch of 500 or more signatures (under a same signing key) with a single ring multiplication.

We do not provide the first two optimizations, since they are prohibited by the submission. We also do not provide the third optimizations for conservative purpose. The fourth optimization is out of scope of this submission.

Potential improvement not in this submission package. There are also three potential improvements that we are aware of, but are not included in this submission.

1. A better Gaussian sampler.
2. A security argument against a quantum random oracle.
3. Efficient Number Theoretic Transform (NTT).
4. Hoffman encoding on Gaussian vectors.

The first two items are active research area. We believe that we shall see many improvements from the PQC community and it is too early to fix on a single solution. We shall include those improvements once they are available, and when a minor revision is allowed. Nonetheless, we remark that our Box-Muller based Gaussian sampler is already quite efficient, and has previously been used in the literature, for example in HElib [13].

We did not implement item 3 due to time constrains. Our naive NTT algorithm takes roughly $O(N^2/2)$ operations where N is the degree of the polynomial. We are aware of the Cooley-Tukey method which runs in $O(N \log N)$

time, and improves signing speed up to 10 times in practice. This improvement will potentially increase the key generation speed; our signing and verification algorithms does not use NTT. We are willing to provide implementation during the revision phrase.

The 4th item is a well known algorithm. It will improve the signature size when Gaussian sampling is used. However, a side effect is that the length of the signatures will be a variable and thus make it difficult to deal with the provided API. Nonetheless, in practice it is suggested that using this encoding will lead to the best performance.

3.8 Known Answer Test Values

A set of KAT values are provided in the KAT folder.

4 IPR Statement

All the related statements are provided in the statement folder.

References

1. Erdem Alkim, Nina Bindel, Johannes A. Buchmann, and Özgür Dagdelen. TESLA: tightly-secure efficient signatures from standard lattices. *IACR Cryptology ePrint Archive*, 2015:755, 2015.
2. Erdem Alkim, Léo Ducas, Thomas Pöppelmann, and Peter Schwabe. Post-quantum key exchange - A new hope. In *25th USENIX Security Symposium, USENIX Security 16, Austin, TX, USA, August 10-12, 2016.*, pages 327–343, 2016.
3. Joël Alwen, Stephan Krenn, Krzysztof Pietrzak, and Daniel Wichs. Learning with rounding, revisited - new reduction, properties and applications. In *Advances in Cryptology - CRYPTO 2013 - 33rd Annual Cryptology Conference, Santa Barbara, CA, USA, August 18-22, 2013. Proceedings, Part I*, pages 57–74, 2013.
4. Leon Groot Bruinderink, Andreas Hülsing, Tanja Lange, and Yuval Yarom. Flush, gauss, and reload - A cache attack on the BLISS lattice-based signature scheme. In *Cryptographic Hardware and Embedded Systems - CHES 2016 - 18th International Conference, Santa Barbara, CA, USA, August 17-19, 2016, Proceedings*, pages 323–345, 2016.
5. Yuanmi Chen and Phong Q Nguyen. BKZ 2.0: Better lattice security estimates. In *ASIACRYPT 2011*, pages 1–20. Springer, 2011.
6. Wei Dai, Berk Sunar, John M. Schanck, William Whyte, and Zhenfei Zhang. NTRU modular lattice signature scheme on CUDA gpus. In *International Conference on High Performance Computing & Simulation, HPCS 2016, Innsbruck, Austria, July 18-22, 2016*, pages 501–508, 2016.
7. Wei Dai, William Whyte, and Zhenfei Zhang. Optimizing polynomial convolution for ntruencrypt. TBA.
8. Léo Ducas, Alain Durmus, Tancrède Lepoint, and Vadim Lyubashevsky. Lattice signatures and bimodal gaussians. In *Advances in Cryptology - CRYPTO 2013 - 33rd Annual Cryptology Conference, Santa Barbara, CA, USA, August 18-22, 2013. Proceedings, Part I*, pages 40–56, 2013.

9. Léo Ducas, Tancrède Lepoint, Vadim Lyubashevsky, Peter Schwabe, Gregor Seiler, and Damien Stehlé. CRYSTALS - dilithium: Digital signatures from module lattices. *IACR Cryptology ePrint Archive*, 2017:633, 2017.
10. Léo Ducas and Phong Q. Nguyen. Learning a zonotope and more: Cryptanalysis of ntrusign countermeasures. In *Advances in Cryptology - ASIACRYPT 2012 - 18th International Conference on the Theory and Application of Cryptology and Information Security, Beijing, China, December 2-6, 2012. Proceedings*, pages 433–450, 2012.
11. Nicolas Gama, Phong Q. Nguyen, and Oded Regev. Lattice enumeration using extreme pruning. In *EUROCRYPT 2010*, volume 6110 of *LNCS*, pages 257–278. Springer, 2010.
12. Oded Goldreich, Shafi Goldwasser, and Shai Halevi. Public-key cryptosystems from lattice reduction problems. In *Advances in Cryptology - CRYPTO '97, 17th Annual International Cryptology Conference, Santa Barbara, California, USA, August 17-21, 1997, Proceedings*, pages 112–131, 1997.
13. Shai Halevi and Victor Shoup. Algorithms in helib. In *Advances in Cryptology - CRYPTO 2014 - 34th Annual Cryptology Conference, Santa Barbara, CA, USA, August 17-21, 2014, Proceedings, Part I*, pages 554–571, 2014.
14. Jeffrey Hoffstein, Nick Howgrave-Graham, Jill Pipher, Joseph H. Silverman, and William Whyte. NTRUSIGN: digital signatures using the NTRU lattice. In *Topics in Cryptology - CT-RSA 2003, The Cryptographers' Track at the RSA Conference 2003, San Francisco, CA, USA, April 13-17, 2003, Proceedings*, pages 122–140, 2003.
15. Jeffrey Hoffstein, Jill Pipher, John M. Schanck, Joseph H. Silverman, and William Whyte. Transcript secure signatures based on modular lattices. In *Post-Quantum Cryptography - 6th International Workshop, PQCrypto 2014, Waterloo, ON, Canada, October 1-3, 2014. Proceedings*, pages 142–159, 2014.
16. Jeffrey Hoffstein, Jill Pipher, John M. Schanck, Joseph H. Silverman, William Whyte, and Zhenfei Zhang. Choosing parameters for ntruencrypt. In *Topics in Cryptology - CT-RSA 2017 - The Cryptographers' Track at the RSA Conference 2017, San Francisco, CA, USA, February 14-17, 2017, Proceedings*, pages 3–18, 2017.
17. Jeffrey Hoffstein, Jill Pipher, William Whyte, and Zhenfei Zhang. A signature scheme from learning with truncation. Cryptology ePrint Archive, Report 2017/995, 2017. http://eprint.iacr.org/2017/995.
18. Nick Howgrave-Graham. *Advances in Cryptology - CRYPTO 2007: 27th Annual International Cryptology Conference, Santa Barbara, CA, USA, August 19-23, 2007. Proceedings*, chapter A Hybrid Lattice-Reduction and Meet-in-the-Middle Attack Against NTRU, pages 150–169. Springer Berlin Heidelberg, Berlin, Heidelberg, 2007.
19. Vadim Lyubashevsky. Fiat-Shamir with aborts: applications to lattice and factoring-based signatures. In *Advances in cryptology—ASIACRYPT 2009*, volume 5912 of *Lecture Notes in Comput. Sci.*, pages 598–616. Springer, Berlin, 2009.
20. Vadim Lyubashevsky. Fiat-shamir with aborts: Applications to lattice and factoring-based signatures. In *Advances in CryptologyASIACRYPT 2009*, page 598616. Springer, 2009.
21. Vadim Lyubashevsky. Lattice signatures without trapdoors. In David Pointcheval and Thomas Johansson, editors, *EUROCRYPT 2012*, volume 7237 of *LNCS*, pages 738–755. Springer, 2012.
22. Phong Q. Nguyen and Oded Regev. Learning a parallelepiped: Cryptanalysis of GGH and NTRU signatures. *J. Cryptology*, 22(2):139–160, 2009.
