# Supplementary material for: Research on blockchain smart contract technology based on resistance to quantum computing attacks
Source: PLoS One. 2024 May 23;19(5):e0302325. doi: 10.1371/journal.pone.0302325 (PMC11115269; doi:10.1371/journal.pone.0302325)
Supplement: S1 File — (ZIP) [file pone.0302325.s001.zip › data/ntru2/pqntrusign-nist-submission-rev/supporting document/spec.pdf]

# Efficient Embedded Security Standards (EESS)

## EESS #1: Implementation Aspects of NTRUEncrypt and pqNTRUSign

Consortium for Efficient Embedded Security

August 31, 2017

Version 3.3

© Consortium for Efficient Embedded Security
License to copy this document is granted provided it is identified as
"Efficient Embedded Security Standards (EESS) #1" in all material referencing it.

Comments regarding this document are welcomed by the editor,
William Whyte, eess-editor@securityinnovation.com.

# Table of Contents

1 Overview 7
1.1 Scope 7
1.2 Purpose 7
1.3 Compliance 7
1.4 EESS Publication Guidelines 8
1.5 Intellectual Property 8
2 Normative references 8
3 Definitions, acronyms and abbreviations 8
3.1 Definitions 8
3.2 Acronyms and abbreviations 14
4 Types of cryptographic techniques 15
4.1 General model 15
4.2 Schemes 15
4.3 Random Polynomial Generation methods 17
4.4 Aux functions 18
4.5 Algorithm specification conventions 18
5 Mathematical notation 18
6 Premileries on polynomials and rings 21
6.1 Introduction 21
6.2 Common polynomial operations and conversions 21
6.2.1 Polynomial representation and conversion 21
6.2.1.1 Integer form 21
6.2.1.2 NTT form 21
6.2.1.3 Conversions 22
6.2.2 Norms of a polynomial 22
6.2.2.1 Infinity Norm 22
6.2.2.2 Euclidean norm 22
6.2.3 Polynomial generation 22
6.2.3.1 Uniform mod q polynomial generator 22
6.2.3.2 Fixed weight binary polynomial generator 23
6.2.3.3 Fixed weight trinary polynomial generator 23
6.2.3.4 Discrete Gaussian polynomial generator 24

6.2.3.4.1 Discrete Gaussian sampler over the integers 24
6.3 Polynomial arithmetics over the integer domain 25
6.3.1 Polynomial multiplication 25
6.3.2 Inversion in a polynomial ring 25
6.3.2.1 The Polynomial Division Algorithm in $Z_p[X]$ 25
6.3.2.2 The Extended Euclidean Algorithm in $Z_p[X]$ 25
6.3.2.3 Inverses in $Z_p[X]/(X^N – 1)$ 26
6.3.2.4 Inverses in $Z_{p^\wedge r}[X]/(X^N – 1)$ 26
6.4 Polynomial arithmetics over NTT 27
7 Data Types and Conversions 27
7.1 Bit Strings and Octet Strings 27
7.2 Converting Between Integers and Bit Strings (I2BSP and BS2IP) 28
7.2.1 Integer to Bit String Primitive (I2BSP) 28
7.2.2 Bit String to Integer Primitive (BS2IP) 28
7.3 Converting Between Integers and Octet Strings (I2OSP and OS2IP) 28
7.3.1 Integer to Octet String Primitive (I2OSP) 28
7.3.2 Octet String to Integer Primitive (OS2IP) 29
7.4 Converting Between Bit Strings and Right-Padded Octet Strings (BS2ROSP and ROS2BSP) 29
7.4.1 Bit String to Right-Padded Octet String Primitive (BS2ROSP) 29
7.4.2 Right-Padded Octet String to Bit String Primitive (ROS2BSP) 29
7.5 Converting Between Ring Elements and Bit Strings (RE2BSP and BS2REP) 30
7.5.1 Ring Element to Bit String Primitive (RE2BSP) 30
7.5.2 Bit String to Ring Element Primitive (BS2REP) 30
7.6 Converting Between Ring Elements and (Compact) Octet Strings (RE2OSP and OS2REP) 31
7.6.1 Ring Element to Octet String Primitive (RE2OSP) 31
7.6.2 Octet String to Ring Element Primitive (OS2REP) 31
7.7 Converting Between Ring Elements and Padded Octet Strings (RE2POSP and POS2REP) 31
7.7.1 Ring Element to Padded Octet String Primitive (RE2POSP) 31
7.7.2 Octet String to Ring Element Primitive (OS2REP) 32
7.8 Converting Between Trinary Ring Elements and Octet Strings (TRE2OSP and OS2TREP) 32
7.8.1 Trinary Ring Element to Octet String Primitive (TRE2OSP) 32

7.8.2 Octet String to Trinary Ring Element Primitive (OS2TREP) 33
8 Supporting algorithms 33
8.1 Overview 33
8.2 Hash Functions and XOFs 33
9 NTRU-KEM 34
9.1 KEM Scheme Operations 34
9.1.1 Key Generation 34
9.1.2 Encapsulation operations 34
9.1.3 Decryption Operation 35
9.1.4 Key Pair Validation Methods 35
9.1.4.1 Key Pair Validation for Trinary Keys 35
9.1.5 Public-key validation 36
9.1.5.1 Full public-key validation 36
9.1.5.2 Partial public-key validation and plausibility tests 36
9.1.5.2.1 Overview 36
9.1.5.2.2 Example suite of plausibility tests 36
9.2 Proposed Parameter Sets 37
10 NTRU-CCA 37
10.1 Encryption Scheme Operations 37
10.1.1 Key Generation 37
10.1.2 Encryption Operation 37
10.1.3 Decryption Operation 38
10.1.4 Key Pair Validation Methods 39
10.1.5 Public-key validation 39
10.2 Possible Parameter Sets 39
11 SS-NTRU-KEM 40
11.1 SS-KEM Scheme Operations 40
11.1.1 Key Generation 40
11.1.2 Encapsulation operations 41
11.1.3 Decryption Operation 41
11.1.4 Key Pair Validation Methods 41
11.1.4.1 Key Pair Validation for Gaussian Keys 42
11.1.5 Public-key validation 42
11.1.5.1 Full public-key validation 42

11.1.5.2 Partial public-key validation and plausibility tests 42
11.1.5.2.1 Overview 42
11.1.5.2.2 Example suite of plausibility tests 43
11.2 Proposed Parameter Sets 43
12 SS-NTRU-CCA 43
12.1 Encryption Scheme Operations 43
12.1.1 Key Generation 44
12.1.2 Encryption Operation 44
12.1.3 Decryption Operation 44
12.1.4 Key Pair Validation Methods 45
12.1.5 Public-key validation 46
12.2 Possible Parameter Sets 46
13 Digital signature scheme 46
13.1 Signature Scheme Operations 46
13.1.1 Key Generation 46
13.1.2 Signing Operation 47
13.1.3 Verification Operation 48
13.1.4 Key Pair Validation Methods 48
13.1.5 Public-key validation 48
13.2 Possible Parameter Sets 49
13.2.1 General 49
14 ASN.1 Syntax 49
14.1 General Types 49
14.1.1 General Vector Types 49
14.2 ASN.1 for NTRUEncrypt SVES 51
14.2.1 NTRUEncrypt Public Keys 52
14.2.2 NTRUEncrypt Private Keys 52
14.2.3 NTRUEncrypt Encrypted Data 53
14.2.4 NTRUEncrypt Parameters 54
14.3 ASN.1 for pqNTRUSign 54
14.3.1 pqNTRUSign Public Keys 55
14.3.2 pqNTRUSign Private Keys 56
14.3.3 pqNTRUSign Signature Data 56
14.3.4 pqNTRUSign Parameters 57

Appendix A - NTRU ASN.1 Module 58
Appendix B - Security Considerations 62
B.1 Security against classical computers 62
B.2 Security against quantum computers 62
B.3 Rejection sampling for lattice based signatures 63
Appendix B - Test Vectors 64
Appendix C - Revision History 64
Appendix D – References 64

# 1 Overview

## 1.1 Scope

This document specifies common techniques and implementation choices for

- Key encapsulation mechanisms:
  - NTRU-KEM: the NTRU public-key cryptography algorithms
  - SS-NTRU-KEM: the NTRU public-key cryptography algorithms with parameters derived from \cite;
- Public key encryption algorithms:
  - NTRU-CCA: the NTRU public-key cryptography algorithms that is secure against chosen ciphertext attacks;
  - SS-NTRU-CCA: the NTRU public-key cryptography algorithms that is secure against chosen ciphertext attacks, with parameters derived from \cite{};
- Digital signature schemes
  - pqNTRUSign: the modular lattice digital signature scheme instantiated with NTRU lattices.

Topics covered include:

- Cryptographic primitives: The building blocks for a secure cryptographic scheme
- Cryptographic schemes: Complete sequences of operations for performing secure cryptographic functions
- Parameter choices: Specific selections of approved sets of values for cryptographic parameters
- ASN.1 syntax : Standard formats of cryptographic data items

In addition, this standard includes relevant information to assist in the development and interoperable implementation, including security estimation.

## 1.2 Purpose

Enormous investments in wireless and consumer infrastructures mandate the need for stronger, more efficient security. First-generation security solutions offer inadequate efficiency and scalability to meet the requirements of mass-market adoption of wireless and embedded consumer applications. To address this need, new security infrastructures are emerging and must be carefully, but rapidly, defined.

In order to ensure interoperability within wired and wireless environments and allow for the rapid deployment of emerging security infrastructures, the Consortium for Efficient Embedded Security (CEES) began work on the Efficient Embedded Security Standards (EESS) in order to provide universal specifications for creating secure, interoperable implementations of highly efficient, highly scalable public-key security.

CEES intends that the EESS will combine the experience and knowledge of experts in academia as well as in commercial industry to provide a complete specification of well-studied, efficient and interoperable methodologies using modern public-key techniques. EESS #1 is designed to specify highly efficient public-key cryptographic techniques that can be used in highly scalable secure applications.

## 1.3 Compliance

Implementations may claim compliance with the cryptographic schemes included in this standard provided the external interface (input and output) to the schemes is identical to the interface specified in this document and the supported encoding methods and parameter selections are used. Internal computations may be performed as specified in this document, or may be performed via an equivalent sequence of

operations. In this document, the word "shall" implies a requirement for an implementation to meet the standard, while the word "should" denotes a choice left to the implementer.

### 1.4 EESS Publication Guidelines

CEES maintains control over the contents and publication of the EESS series. This document will be regularly updated via the NTRU Open Source repository on github, https://github.com/NTRUOpenSourceProject/ntru-crypto, and other means as appropriate. In addition, the Consortium welcomes input from the community at large. Comments may be submitted to the editor, William Whyte, at eess-editor@securityinnovation.com.

### 1.5 Intellectual Property

Compliance with this standard, any other CEES standard, or any standard referenced herein may be subject to intellectual property claims by third parties. By publication of this document, CEES takes no position with respect to the validity of such claims. When possible, the CEES has made efforts to obtain information relating to patent coverage of techniques included in EESS.

Security Innovation Inc. has released Patents related to NTRUEncrypt to the public domain under CC0 license:

| Invention | U.S. Patent No. | U.S. Issue Date | U.S. Application No. | U.S. Filing Date | Countries |
|---|---|---|---|---|---|
| Public key cryptosystem method and apparatus | 6,081,597 | 27-Jun-00 | 08/914,449 | 19-Aug-97 | US, Japan, Canada, Australia, Israel, China, Europe |
| Ring-based public key cryptosystem method | 6,298,137 | 2-Oct-01 | 09/543,708 | 5-Apr-00 | US, Europe, Canada |
| Speed enhanced cryptographic method and apparatus | 7,031,468 | 18-Apr-06 | 09/939,531 | 24-Aug-01 | US, Europe, Canada |

Onboard Security Inc. has been assigned ownership of U.S. Patent Application No. 20150229478, which covers aspects of the pqNTRUSign digital signature scheme. In addition, Onboard Security Inc. may have applied for additional patent coverage on implementation techniques defined in this standard.

## 2 Normative references

The following referenced documents are indispensable for the application of this document (i.e., they must be understood and used, so each referenced document is cited in text and its relationship to this document is explained). For dated references, only the edition cited applies. For undated references, the latest edition of the referenced document (including any amendments or corrigenda) applies.

1. FIPS 180, *Secure Hash Standard*, Federal Information Processing Standards Publication 180, U.S. Department of Commerce/National Institute of Standards and Technology, National Technical Information Service, Springfield, Virginia.[1]

## 3 Definitions, acronyms and abbreviations

### 3.1 Definitions

For the purposes of this standard, the following terms and definitions apply. *The Authoritative Dictionary of IEEE Standards, Seventh Edition,* should be referenced for terms not defined in this clause.

**3.1.1 Algorithm:** A clearly specified mathematical process for computation; a set of rules which, if followed, give a prescribed result.

[1] FIPS 180 current version as of 2016 is FIPS 180-4, March, 2012, available at http://csrc.nist.gov/publications/fips/fips180-4/fips-180-4.pdf.

**3.1.2 Asymmetric Cryptographic Algorithm:** A cryptographic algorithm that uses two related keys, a public key and a private key; the two keys have the property that, given the public key, it is computationally infeasible to derive the private key.

**3.1.3 Authentication (of a message):** The act of determining that a message has not been changed since leaving its point of origin. The identity of the originator is implicitly verified.

**3.1.4 Authentication of Ownership:** The assurance that a given, identified party intends to be associated with a given public key. May also include assurance that the party possesses the corresponding private key (see IEEE Std 1363-2000, Annex D.3.2, for more information).

**3.1.5 Big Modulus:** The big modulus $q$ is used to define the larger polynomial ring. The modulus $q$ can generally be taken to be any value that is relatively prime in the ring to the small modulus p.

**3.1.6 Birthday Paradox:** For a category size of 365 (the days in a year), after only 23 people are gathered, the probability is greater than 0.5 that at least two people have a common birthday (month and day). The reason is that among 23 people, there are $23 \times (23-1)/2 = 253$ pairs of people, each with a 1/365 chance of having matching birthdays. The chance of no matching birthday is therefore $(364/365)^{253} \sim 0.4995$. In general, any case where the criterion for success is to find a collision (two matching values) rather than a hit (one value which matches a pre-selected one) displays this pairing property, so that the size of the space to be searched for success is about the square root of the size of the space of all possible values.

**3.1.7 Bit Length:** See: length.

**3.1.8 Bit String:** An ordered sequence of 0's and 1's. The left-most bit is the most-significant bit of the string. The right-most bit is the least-significant bit of the string. A bit and a bit string of length 1 are equivalent for all purposes of this standard.

**3.1.9 Blinding Polynomial:** In this standard, the ciphertext $e$ is generated according to the equation $e = r \times h + m'$, where $h$ is the public key, $m'$ is the message representative, and r is a pseudorandomly generated "blinding polynomial"

**3.1.10 Blinding Polynomial Generation Methods:** In the encryption schemes in this document, a blinding polynomial generation method (LBP-BPGM) is used to generate a blinding polynomial r from the padded message *pm* in order to provide plaintext awareness.

**3.1.11 Blinding Polynomial Space:** The space that a LBP-BPGM selects from. Usually defined implicitly by the definition of the LBP-BPGM.

**3.1.12 Certificate:** The public key and identity of an entity together with some other information rendered unforgeable by signing the certificate with the private key of the certifying authority, which issued that certificate.

**3.1.13 Ciphertext:** The result of applying encryption to a message. Contrast: plaintext. See also: encryption.

**3.1.14 Composite:** An integer which has at least two prime factors.

**3.1.15 Confidentiality:** The property that information is not made available or disclosed to unauthorized individuals, entities, or processes.

**3.1.16 Conformance Region:** A set of inputs to a primitive or a scheme operation for which an implementation operates in accordance with the specification of the primitive or scheme operation

**3.1.17 Cryptographic Family:** A set of cryptographic techniques in similar mathematical settings. For example, this standard presents a single family of techniques based on the underlying hard problems of finding a short vector and a close vector in a lattice.

**3.1.18 Cryptographic Hash Function:** See hash function.

**3.1.19 Cryptographic Key (Key):** A parameter that determines the operation of a cryptographic function such as: the transformation from plain text to cipher text and vice versa; synchronized generation of keying material; digital signature computation or validation.

**3.1.20 Cryptography:** The discipline which embodies principles, means and methods for the transformation of data in order to hide its information content, prevent its undetected modification, prevent its unauthorized use or a combination thereof.

**3.1.21 Data Integrity:** A property whereby data has not been altered or destroyed.

**3.1.22 Decrypt:** To produce plaintext (readable) from ciphertext (unreadable). Contrast: encrypt. See also: ciphertext; encryption; plaintext.

**3.1.23 Dimension:** The dimension $N$ identifies the dimension of the convolution polynomial ring used. The dimension of the associated lattice problem is $2N$. Elements of the ring are represented as polynomials of degree $N - 1$.

**3.1.24 Domain Parameters:** A set of mathematical objects, such as fields or groups, and other information, defining the context in which public/private key pairs exist. More than one key pair may share the same domain parameters. Not all cryptographic families have domain parameters. See also: public/private key pair; valid domain parameters.

**3.1.25 Domain Parameter Validation:** The process of ensuring or verifying that a set of domain parameters is valid. See also: domain parameters; key validation; valid domain parameters.

**3.1.26 Encrypt:** To produce ciphertext (unreadable) from plaintext (readable). Contrast: decrypt. See also: ciphertext; encryption; plaintext.

**3.1.27 Encryption Primitives:** An operation that converts a plaintext to a ciphertext, providing security according to the difficulty of solving an underlying hard problem, against a ciphertext-only attack by a passive attacker who only has a single non-chosen ciphertext. A building block for encryption schemes.

**3.1.28 Encryption Scheme:** A means for providing encryption, based on an encryption primitive, that is secure against both active and passive attackers. A secure encryption scheme typically provides semantic security (an attacker who knows that one of two messages has been encrypted will find it computationally infeasible to determine which) against an attacker who can make polynomially many queries to a decryption oracle.

**3.1.29 Entity:** A participant in any of the schemes in this standard. The words “entity” and “party” are used interchangeably. This definition may admit many interpretations: it may or may not be limited to the necessary computational elements; it may or may not include or act on behalf of a legal entity. The particular interpretation chosen does not affect operation of the key agreement schemes.

**3.1.30 Exclusive OR:** A mathematical bit-wise operation, symbol $\oplus$, defined as:

$0 \oplus 0 = 0$,

$0 \oplus 1 = 1$,

$1 \oplus 0 = 1$, and

$1 \oplus 1 = 0$.

Equivalent to binary addition without carry. May also be applied to bit strings: the XOR of two bit strings of equal length is the concatenation of the XORs of the corresponding elements of the bit strings.

**3.1.31 Family:** See: cryptographic family.

**3.1.32 Field:** A setting in which the usual mathematical operations (addition, subtraction, multiplication, and division by nonzero quantities) are possible and obey the usual rules (such as the commutative, associative, and distributive laws).

**3.1.33 Finite Field:** A field in which there are only a finite number of quantities.

**3.1.34 First Bit:** The leading bit of a bit string or an octet. For example, the first bit of 0110111 is 0. Contrast: last bit. Syn: most significant bit; leftmost bit. See also: bit string; octet.

**3.1.35 First Octet:** The leading octet of an octet string. For example, the first octet of 1c 76 3b e4 is 1c. Contrast: last octet. Syn: most significant octet; leftmost octet. See also: octet; octet string.

**3.1.36 Hash Function:** A function which maps a bit string of arbitrary length to a fixed-length bit string and satisfies the following properties:

1. It is computationally infeasible to find any input which maps to any pre-specified output;
2. It is computationally infeasible to find any two distinct inputs which map to the same output.

**3.1.37 Hash Value**: The result of applying a hash function to a message.

**3.1.38 Key:** See cryptographic key.

**3.1.39 Key Confirmation:** The assurance of the legitimate participants in a key establishment protocol that the intended recipients of the shared key actually possess the shared key.

**3.1.40 Key Derivation:** The process of deriving one or more session keys from a shared secret and (possibly) other, public information. Such a function can be constructed from a one-way hash function such as SHA-2.

**3.1.41** Key Exchange algorithm: A protocol that reveals a secret key to its legitimate participants for cryptographic use.

**3.1.42 Key** Encapsulation mechanism**:** A key establishment protocol under which the secret key is determined by the initiating party.

**3.1.43 Key Generation Primitive:** A method used to generate a key pair.

**3.1.44 Key Management:** The generation, storage, secure distribution and application of keying material in accordance with a security policy.

**3.1.45 Key Pair:** When used in public key cryptography, a private key and its corresponding public key. The public key is commonly available to a wide audience and can be used to encrypt messages or verify digital signatures; the private key is held by one entity and not revealed to anyone--it is used to decrypt messages encrypted with the public key and/or produce signatures that can verified with the public key. A public/private key pair can also be used in key agreement. In some cases, a public/private key pair can only exist in the context of domain parameters. See also: digital signature; domain parameters; encryption; key agreement; public-key cryptography; valid key; valid key pair.

**3.1.46 Key Validation:** The process of ensuring or verifying that a key conforms to the arithmetic requirements for such a key in order to thwart certain types of attacks. See also: domain parameter validation; public/private key pair; valid key; valid key pair.

**3.1.47 Keying Material:** The data (e.g., keys, certificates and initialization vectors) necessary to establish and maintain cryptographic keying relationships.

**3.1.48 Last Bit:** The trailing bit of a bit string or an octet. For example, the last bit of 0110111 is 1. Contrast: first bit. Syn: least significant bit; rightmost bit. See also: first bit; octet.

**3.1.49 Last Octet:** The trailing octet of an octet string. For example, the last octet of 1c 76 3b e4 is e4. Contrast: first octet. Syn: least significant octet; rightmost octet. See also: octet; octet string.

**3.1.50 Least Significant:** See: last bit; last octet.

**3.1.51 Leftmost Bit:** See: first bit.

**3.1.52 Leftmost Octet:** See: first octet.

**3.1.53 Length:** (1) Length of a bit string is the number of bits in the string. (2) Length of an octet string is the number of octets in the string. (3) Length in bits of a nonnegative integer $n$ is $\lceil$log2 ($n$ + 1)$\rceil$ (i.e., the number of bits in the integer's binary representation). (4) Length in octets of a nonnegative integer $n$ is $\lceil$log256 ($n$ + 1)$\rceil$ (i.e., the number of digits in the integer's representation base 256). For example, the length in bits of the integer 500 is 9, and its length in octets is 2.

**3.1.54 Message Authentication Code (MAC):** A cryptographic value which is the results of passing a financial message through the message authentication algorithm using a specific key.

**3.1.55 Message Length Encoding Length:** In SVES, the length of the message that is to be encrypted is encoded in the padded message. The length of the field that represents the length of the message, called the message length encoding length, is represented by the parameter *lLen*. For all parameter sets in this document *lLen* is set to 1.

**3.1.56 Message Representative:** A mathematical value for use in a cryptographic primitive, computed from a message that is input to an encryption or a digital signature scheme and uniquely linked to that message. See also: encryption scheme; digital signature scheme.

**3.1.57 Modular Lattice:** A lattice in which (among other things) all values are integers reduced mod $q$.

**3.1.58 Most Significant:** See: first bit; first octet.

**3.1.59 Norm:** A measure of the "size" of a vector or polynomial.

**3.1.60 Octet:** A bit string of length 8. An octet has an integer value between 0 and 255 when interpreted as a representation of an integer in base 2. An octet can also be represented by a hexadecimal string of length 2, where the hexadecimal string is the representation of its integer value base 16. For example, the integer value of the octet 10011101 is 157; its hexadecimal representation is 9d. Also commonly known as a byte. See also: bit string.

**3.1.61 Octet String:** An ordered sequence of octets. See also: octet.

**3.1.62 Owner:** The entity whose identity is associated with a key pair.

**3.1.63 Parameters:** See: domain parameters.

**3.1.64 Plaintext:** A message before encryption has been applied to it; the opposite of ciphertext. Contrast: ciphertext. See also: encryption.

**3.1.65 Prime Number:** A positive integer that is greater than 1 and divisible only by 1 and itself.

**3.1.66 Primitives:** Cryptographic primitives used in the a public key encryption scheme include key generation primitives, encryption primitives and decryption primitives; that used in key encapsulation scheme include key generation primitives, encapsulation primitives and decapsulation primitives; that used in public key digital signature primitives include key generation primitives, signing primitives and verification primitives.

**3.1.67 Private Key:** The private element of the public/private key pair. See also: public/private key pair; valid key.

**3.1.68 Private Key Space:** The space from which a key generation primitive selects the private key.

**3.1.69 Public Key:** The public element of the public/private key pair. See also: public/private key pair; valid key.

**3.1.70 Public-key Cryptography:** Methods that allow parties to communicate securely without having prior shared secrets through the use of public/private key pairs. Contrast: symmetric cryptography. See also: public/private key pair.

**3.1.71 Public Key Space:** The space from which a key generation primitive selects the public key.

**3.1.72 Public Key Validation:** See key validation.

**3.1.73 Public/Private Key Pair:** See key pair.

**3.1.74** **Salt:** Random bits that are used to pad the message during encryption, to provide for semantic security.

**3.1.75** **Salt Size:** The size of the salt. Can be expressed in bits or octets.

**3.1.76** **Rightmost Bit:** See: last bit.

**3.1.77** **Rightmost Octet:** See: last octet.

**3.1.78** **Ring:** A setting in which addition, subtraction, and multiplication are possible, and division by a given nonzero quantity may or may not be possible. A field is a special case of a ring. See also: field.

**3.1.79** **Ring Element:** In general, an element in a ring. In the context of this standard, a *binary N-ring element* refers to an element in the ring $(\mathbf{Z}/2\mathbf{Z})[X]/(X^N - 1)$, which is to say a binary polynomial of degree $N$-1 or an array of $N$ binary elements. A $(q, N)$*-ring element* refers to an element in the ring $(\mathbf{Z}/q\mathbf{Z})[X]/(X^N - 1)$, which is to say a polynomial of degree $N$-1 with coefficients reduced mod $q$ or an array of $N$ elements each taken mod $q$.

**3.1.80** **Scheme Options:** Scheme options consist of parameters and algorithms that do not affect the crucial space (i.e. that are not domain parameters), but that shall be agreed upon in order to implement the encryption scheme.

**3.1.81** **Secret Key:** A key used in symmetric cryptography; needs to be known to all legitimate participating parties involved, but cannot be known to an adversary. Contrast: public/private key pair. See also: key agreement; shared secret key; symmetric cryptography.

**3.1.82** **Secret Value:** A value that can be used to derive a secret key, but typically cannot by itself be used as a secret key. See also: secret key.

**3.1.83** **Shared Secret Key:** A secret key shared by two parties, usually derived as a result of a key agreement scheme. See also: key agreement; secret key.

**3.1.84** **Shared Secret Value:** A secret value shared by two parties, usually during a key agreement scheme. See also: key agreement; secret value.

**3.1.85** **Signature:** See: digital signature.

**3.1.86** **Small Modulus:** In LBP-PKE, the small modulus $p$ is used for key generation and for modular reduction during decryption.

**3.1.87** **Statistically Unique:** For the generation of $n$-bit quantities, the probability of two values repeating is less than or equal to the probability of two $n$-bit random quantities repeating. More formally, an element chosen from a finite set $S$ of $n$ elements is said to be "statistically unique" if the process that governs the selection of this element is such that, for any integer $L \leq n$, the probability that all of the first $L$ selected elements are different is no smaller than the probability of this happening when the elements are drawn uniformly randomly from $S$.

**3.1.88** **Symmetric Cryptographic Algorithm:** A cryptographic algorithm that uses one cryptographic key. Anyone who knows the key can both encrypt and decrypt a message, and can calculate a Message Authentication Code using that key.

**3.1.89** **Symmetric Cryptography:** Methods that allow parties to communicate securely only when they already share some prior secrets, such as the secret key. Contrast: public-key cryptography. See also: secret key.

**3.1.90** **Symmetric Key:** A cryptographic key that is used in symmetric cryptographic algorithms. The same symmetric key that is used for encryption is also used for decryption.

**3.1.91** **User:** A party that uses a public key.

**3.1.92 Valid Domain Parameters:** A set of domain parameters that satisfies the specific mathematical definition for the set of domain parameters of its family. While a set of mathematical objects may have the general structure of a set of domain parameters, it may not actually satisfy the definition (for example, it may be internally inconsistent) and thus not be valid. See also: domain parameters; public/private key pair; valid key; valid key pair; validation.

**3.1.93 Valid Key:** A key (public or private) that satisfies the specific mathematical definition for the keys of its family, possibly in the context of its set of domain parameters. While some mathematical objects may have the general structure of keys, they may not actually lie in the appropriate set (for example, they may not lie in the appropriate subgroup of a group or be out of the bounds allowed by the domain parameters) and thus not be valid keys. See also: domain parameters; public/private key pair; valid domain parameters; valid key pair; validation.

**3.1.94 Valid Key Pair:** A public/private key pair that satisfies the specific mathematical definition for the key pairs of its family, possibly in the context of its set of domain parameters. While a pair of mathematical objects may have the general structure of a key pair, the keys may not actually lie in the appropriate sets (for example, they may not lie in the appropriate subgroup of a group or be out of the bounds allowed by the domain parameters) or may not correspond to each other; such a pair is thus not a valid key pair. See also: domain parameters; public/private key pair; valid domain parameters; valid key; validation.

**3.1.95 Validation:** See: domain parameter validation; key validation.

**3.1.96 Verify:** In relation to a digital signature means to determine accurately: (1) that the digital signature was created by the private key corresponding to the public-key used to verify the signature; and (2) the message has not been altered since its digital signature was created.

**3.1.97** xof: extendable output functions that input an arbitrary length input binary string and output variable/fixed length output string. The secure XOF function used in this standard is SHAKE-256.

## *3.2 Acronyms and abbreviations*

| | |
|---|---|
| BS2IP | Bit String to Integer Conversion Primitive |
| BS2REP | Bit String to Ring Element Conversion Primitive |
| BS2ROSP | Bit String to Right-padded Octet String Conversion Primitive |
| DGS | Discrete Gaussian polynomial generator method |
| DGSi | Discrete Gaussian integer generator method |
| DP | Decryption Primitive |
| DS | Digital signature primitive |
| ES | Encryption Scheme |
| FWBPG | Fixed weight binary polynomial generation method |
| FWTPG | Fixed weight trinary polynomial generation method |
| iNTT | Inverse of number theoretic transformation |
| I2BSP | Integer to Bit String Conversion Primitive |
| I2OSP | Integer to Octet String Conversion Primitive |
| KEM | Key Encapsulation Mechanism primitive |
| KGP | Key Generation Primitive |
| MAC | Message Authentication Code |
| MPM | Message Padding Method |

| | |
|---|---|
| MRGM | Message Representative Generation Method |
| NTRU-KEM | KEM based on NTRU cryptosystem |
| NTRU-CCA | CCA-2 secure PKE based on NTRU cryptosytem |
| NTT | Number theoretic transformation |
| OS2IP | Octet String to Integer Conversion Primitive |
| OS2REP | Octet String to Ring Element Conversion Primitive |
| PKE | Public Key Encryption method |
| RE2BSP | Ring Element to Bit String Conversion Primitive |
| RE2OSP | Ring Element to Octet String Conversion Primitive |
| ROS2BSP | Right-padded Octet String to Bit String Conversion Primitive |
| SS-NTRU | NTRU cryptosystem based on Stehle-Steinfield parameters |
| URPGq | Uniform random mod *q* polynomial generation methods |
| XOF | eXtendable Output Function |

# 4 Types of cryptographic techniques

## 4.1 General model

As stated in Section 1.2, the purpose of this standard is to provide a reference for specifications of a variety of common public-key cryptographic techniques from which applications may select. Different types of cryptographic techniques can be viewed abstractly according to the following three-level general model.

- *Primitives* – basic mathematical operations. Historically, they were discovered based on number-theoretic hard problems. Primitives are not meant to achieve security just by themselves, but they serve as building blocks for schemes.
- *Schemes* – a collection of related operations combining primitives and additional methods (Clause 4.4). Schemes can provide complexity-theoretic security which is enhanced when they are appropriately applied in protocols.
- *Protocols* – sequences of operations to be performed by multiple parties to achieve some security goal. Protocols can achieve desired security for applications if implemented correctly.

From an implementation viewpoint, primitives can be viewed as low-level implementations (e.g., implemented within cryptographic accelerators, or software modules), schemes can be viewed as medium-level implementations (e.g., implemented within cryptographic service libraries), and protocols can be viewed as high-level implementations (e.g., implemented within entire sets of applications).

This standard contains only specifications of schemes.

## 4.2 Schemes

The following types of schemes are defined in this standard:

- Key Encapsulation Mechanism (KEM), in which the initiator, Alice, sends her public key to the receiver Bob; Bob picks a random string as (the seed of) the session key, and encapsulate it with Alice's public key; Alice decapsulate the ciphertext and obtains the session key.
- Public Key Encryption (PKE) schemes, in which any party can encrypt a message using a recipient's public key, and only the recipient can decrypt the message by using its corresponding private key. Encryption schemes may be used for establishing secret keys to be used in symmetric cryptography.

- Digital Signature (DS) schemes, in which only the holder of a secret key can sign a message digest. Anyone can validate the signature using the corresponding public key. Signature schemes may be used for establishing the authenticity of a message or an identity.

Schemes in this standard are presented in a general form based on certain primitives and additional methods. For example, the encryption scheme defined in this standard is based on a key generation primitive, a decryption primitive, and a fixed weight trinary polynomial generation (FWTPG) method; the signature scheme defined in this standard is based on a key generation primitive, a digital signing primitive, a random polynomial generation method (URPG) and/or a discrete Gaussian polynomial generation method (DSG).

Schemes also include key management operations, such as selecting a private key or obtaining another party's public key. For proper security, a party needs to be assured of the true owners of the keys and domain parameters and of their validity. Generation of domain parameters and keys needs to be performed properly, and in some cases validation also needs to be performed. While outside the scope of this standard, proper key management is essential for security.

A *Key Encapsulation Mechanism* scheme is specified by providing the following:

- Name
- Type (e.g. Asymmetric Public-key Encryption Scheme)
- Options (Key Type, Primitives, Parameters)
- Operations
  - Key Pair Generation
  - Key Pair Validation
  - Public Key Validation
  - Encryption Operation
    - Input
    - Output
  - Decryption Operation
    - Input
    - Output

An *Key Encapsulation Mechanism* specification may also include the following:

- Security Considerations
- Implementation Considerations
- Related Standards

A *Public Key Encryption Scheme* is specified by providing the following:

- Name
- Type (e.g. Asymmetric Public-key Encryption Scheme)
- Options (Key Type, Primitives, Parameters)
- Operations
  - Key Pair Generation
  - Key Pair Validation
  - Public Key Validation

  - Encryption Operation
    - Input
    - Output
  - Decryption Operation
    - Input
    - Output

A *Public Key Encryption Scheme* specification may also include the following:

- Security Considerations
- Implementation Considerations
- Related Standards

A *Digital Signature Scheme* is specified by providing the following:

- Name
- Options (Key Type, Primitives, Parameters)
- Operations
  - Key Pair Generation
  - Key Pair Validation
  - Public Key Validation
  - Signing Operation
    - Input
    - Output
  - Verification Operation
    - Input
    - Output

A *Digital Signature Scheme* specification may also include the following:

- Security Considerations
- Implementation Considerations
- Related Standards

The specifications are functional specifications, not interface specifications. As such, the format of inputs and outputs and the procedure by which an implementation of a scheme is invoked are outside the scope of this standard. See section 11 for more information on input and output formats.

## 4.3 Random Polynomial Generation methods

The schemes described in this standard requires generation of random polynomials from a certain distribution. Those methods are sometimes referred to as samplers since they generate samples for a given distribution.
It is assumed that there exists a sufficiently long random binary string which is initiated with a seed of adequate length and obtained via an XOF function such as SHAKE or AES in counter mode. This initial seed is also known as the initial vector in some context.

This string may be generated at the initiation stage, prior to any cryptographic operations; or it may be generated at the run-time by updating the hash digest of the seed.
This string will be the pool of randomness to the following methods. Note that, to enable KAT, it is required that those methods are deterministic, i.e., for a same initial seed, the output polynomials are the same. This standard specifies the following additional methods:

- Uniformly Random modulo $q$ Polynomial Generation Methods (URPG$q$); with special cases:
  - $q = 2$: Uniformly Random Binary Polynomial Generation Methods (URPG$2$);
  - $q = 3$: Uniformly Random Trinary Polynomial Generation Methods (URPG$3$);
- Fixed Weight Random modulo $q$ Polynomial Generation Methods; with special cases:
  - Fixed Weight Random Binary Polynomial Generation Methods (FWBPG);
  - Fixed Weight Random Trinary Polynomial Generation Methods (FWTPG);
- Random Discrete Gaussian distributed Polynomial Generation Methods (DGS).

See Section 6 for more details.

## 4.4 Aux functions

Hash functions are essential to generate blinding polynomials in the encryption scheme and the signature scheme. Blinding polynomial is a polynomial that

- either to blind the randomness to the message during the encryption, or
- to blind the message to its digest.

We require the hash function to be collision resistant and preimage resistant. The recommended hash functions are SHA512 from SHA2 family and SHA3-512 from SHA3 (Keccak) family.

The specified additional methods are required for conformant use of the schemes. The use of an inadequate message encoding method, key derivation function, or auxiliary function may compromise the security of the scheme in which it is used. Therefore, any implementation which chooses not to follow the recommended additional methods for a particular scheme should perform its own thorough security analysis of the resulting scheme.

## 4.5 Algorithm specification conventions

When specifying an algorithm or method, this standard uses four parts to specify different aspects of the algorithm. They are as follows:

**Components**, such as choice of random polynomial generating functions, are parameters that are specified before the beginning of the operation and that are not specific to the particular algorithm call. Components tend to be kept fixed for multiple users and multiple instances of the algorithm call and need not be explicitly specified if they are implicitly known (e.g. if they are defined within a selected object identifier (OID)).

**Inputs**, such as keys and messages, are values that shall be specified for each algorithm call.

**Outputs**, such as ciphertext, are the result of transformations on the inputs.

**Operations** specify the transformations that are performed on the data to arrive at the output. Throughout the standard, the operations are defined as a sequence of steps. A conformant implementation may perform the operations using any sequence of steps that consistently produces the same output as the sequence in this standard. Caution should be taken to ensure that intermediate values are not revealed, however, as they may compromise the security of the algorithms.

# 5 Mathematical notation

When referring to mathematical objects and data objects in this standard, the following notation is used. Throughout the document, numbers at the end of variable names are used to distinguish different, but related values (e.g. *Dmin1*, *Dmin2*, etc.).

| | |
|---|---|
| 0 | Denotes the integer 0, the bit 0, or the additive identity (the element zero) of a ring |
| 1 | Denotes the integer 1, the bit 1, or the multiplicative identity (the element one) of a ring |
| $\times$ | Indicates the convolution product of two polynomials and is also used to indicate multiplication of integers |
| $\oplus$ or XOR | Exclusive OR function |
| $\|\|$ | Concatenation. $A\|\|B$ is the concatenation of the octet strings $A$ and $B$ where the leading octet of $A$ is the leading octet of $A\|\|B$ and the trailing octet of $B$ is the trailing octet of $A\|\|B$. |
| $\|\|a\|\|_\infty$ | The infinity norm (a.k.a. max norm) of a polynomial $a$, which is the maximum of the absolute value of all it coefficients. |
| $\|\|a\|\|_2$ | The Euclidean norm (a.k.a. l2 norm) of a polynomial $a$, which is the square root of the sum of squares of all it coefficients. |
| := | Initialization. $a := b$ means initialize or set the value of $a$ equal to the value of $b$. |
| | |
| $B_s$ and $B_t$ | An acceptance threshold, used in signature verification to determine whether to accept a signature (the signature will be rejected if its max norm is greater than $q/2 - B_s$ or $q/2 - B_t$). |
| $c$ | Encrypted message representative, a polynomial, computed by an encryption primitive |
| $C$ | Encrypted message, an octet string |
| ceil[.] or □.□ | Ceiling function (i.e. the smallest integer greater than or equal to the contents of [.]) |
| $db$ | The number of random bits used as input for encryption |
| $d_f$ | An integer specifying the number of ones (and minus ones) in the polynomials that comprise the private key value $F$ |
| $d_g$ | An integer specifying the number of ones in the polynomials that comprise the temporary polynomial $g$ (often specified as $dg$) |
| $d_r$ | An integer specifying the number of ones in the binding polynomial $r$ in PKE. |
| $e$ | noise added during the encryption in SS-NTRU |
| $f$ | A polynomial that is calculate by $f$=1+$pF$ |
| $F$ | Secret key for (SS-)NTRU and part of secret of pqNTRUSign |
| floor[.] or □.□ | Floor function (i.e. the largest integer less than or equal to the contents of [.]) |

| | |
|---|---|
| *g* | A temporary polynomial used in the key generation process in (SS-)NTRU, or part of Secret key for pqNTRUSign |
| GCD(*a*, *b*) | Greatest Common Divisor of two non-negative integers *a* and *b* or polynomials *a(X)* and *b(X)*. |
| *h* | Public key |
| Hash( ) | A cryptographic hash function computed on the contents of ( ) |
| *hLen* | Length in octets of a hash value. |
| *i* | An integer |
| *k* | Security level in bits. |
| *m* | The message, an octet string, which is used to derive the session key in KEM, or encrypted in PKE, or digitally signed in DS. |
| *M* | The padded and formatted message representative octet string used during encryption and decryption in (SS-)NTRU-CCA. |
| *m'* | The message representative polynomial which is submitted to the encryption primitive in (SS-)NTRU-CCA. |
| mod *q* | Used to reduce the coefficients of a polynomial into some interval of length *q* |
| mod *p* | Used to reduce a polynomial to an element of the polynomial ring mod *p* |
| *N* | Dimension of the polynomial ring used (i.e. polynomials are up to degree *N*-1) |
| *p* | "Small" modulus, an integer or a polynomial |
| *q* | "Big" modulus, usually an integer |
| *r* | A random polynomial in (SS-)NTRU-KEM, or the encryption binding polynomial (generated from the hash of the padded message *M* in (SS-)NTRU-CCA). |
| *x* | The integer input to or output from integer conversion primitives |
| *X* | The indeterminate used in polynomials |
| XGCD(*a*, *b*) | Extended Greatest Common Divisor algorithm with inputs of two non-negative integers *a* and *b* or polynomials *a(X)* and *b(X)*. |
| ***Z*** | The ring of integers |
| $\boldsymbol{Z}_q$ | The ring of integers mod *q*. |
| | |

# 6 Premileries on polynomials and rings

## 6.1 Introduction

The cryptographic techniques specified in this standard require arithmetic in quotient polynomial rings, also called convolution polynomial rings. Intuitively, these algebraic objects consist of polynomials with integer coefficients. Manipulation of these ring elements is accomplished by polynomial arithmetic modulo a fixed polynomial: $X^N - 1$ or $X^N + 1$ in this standard. Here we enumerate all rings that will be used:

1. $X^{443} - 1 \bmod 2048$
2. $X^{743} - 1 \bmod 2048$
3. $X^{761} - 1 \bmod 65447$
4. $X^{512} + 1 \bmod 65536$
5. $X^{1024} + 1 \bmod 1073750017$

Rings #1 and #2 are used for NTRU key encapsulations and encryptions; Rings #3 and #4 are used for pqNTUSign digital signature scheme; Ring #5 is used for SS-NTRU key encapsulations and encryptions. In the rest of this session, we define three sets of operations that handles polynomials over those rings, namely:

a. Common polynomial operations and conversions;
b. Polynomial arithmetic over the integer domain;
c. Polynomial arithmetic over the number theoretic transformation (NTT) domain.

Operations performed over rings #1 and #2 MUST be from Set a) and b); operations over rings #3, #4 and #5 MAY be performed from any of the three sets.

## 6.2 Common polynomial operations and conversions

### 6.2.1 Polynomial representation and conversion

Typically, in mathematical literature, a polynomial $a$ in $X$ is denoted $a(X)$. In this standard, when the meaning is clear from the context, polynomials $a$ in the variable $X$ are simply denoted by $a$. Further, all polynomials used in this standard have degree $N - 1$, unless otherwise noted.

Throughout this document, polynomials are taken mod $q$, where $q$ is an integer. To reduce a polynomial mod $q$, one simply reduces each of the coefficients independently mod $q$ into the appropriate (specified) interval. For polynomials whose degree are greater than $N$, its representation in the ring $R = Z[X]/F(X)$ is taken as modulo $F(X)$. For example, X^N = 1 mod F(X) for F(X) = x^{N-1} - 1.

#### 6.2.1.1 Integer form

Given a polynomial $a$, a variable denoted $a_i$, where $i$ is an integer, represents the coefficient of $a$ of degree $i$. In other words, the polynomial denoted $a$ represents the polynomial $a(X) = a_0 + a_1X + a_2X^2 + a_3X^3 + \ldots + a_iX^i + \ldots + a_{N-1}X^{N-1}$ in the integer domain.

#### 6.2.1.2 NTT form

For rings #3, #4 and #5 we can perform Number Theoretical Transformation. This optional choice allows one to perform polynomial multiplication over the ring more efficiently. For a polynomial $a(X)$ within an NTT domain R, where ω is the primitive root of the ring $X^N - 1$ or $X^N + 1$, the NTT form of $a(X)$ is an N-dimensional vector $\langle b_0, b_1, \ldots, b_{N-1}\rangle$ where each coefficient is between 0 and q-1.

#### 6.2.1.3 Conversions

To convert a polynomial $a(X)$ into its NTT form $b$, one compute where $b_i = a(\omega^i)$ mod $q$ for each 0<= i < N. To transfer an NTT form polynomial $b$ back to its integer representation, one computes $a(X)$ by setting $a_i = \frac{1}{N}\sum_{j=0}^{N-1} b_j\ \omega^{-i\times j}$ mod $q$ for each 0<= i < N.

### 6.2.2 Norms of a polynomial

#### 6.2.2.1 Infinity Norm

For a polynomial $a(X) = a_0 + a_1X + a_2X^2 + a_3X^3 + \ldots + a_iX^i + \ldots + a_{N-1}X^{N-1}$, the infinity norm, $||a||_\infty$ is the maximum absolute value of any of its coefficients. It can be computed using the following or an equivalent sequence of steps:

a) Set b = 0
b) for (i=0; i<N; i++)
    1) if b < absolute_value($a_i$)
        i) b = absolute_value($a_i$)
c) Return b

#### 6.2.2.2 Euclidean norm

The Euclidean norm $||a||_2$ (a.k.a, $l_2$ norm) is the square root of the sum of the square of all its coefficients. It can be computed using the following or an equivalent sequence of steps:

a) Set $b = 0$
b) for (i=0; i<N; i++)
    1) $b$ += $a_i^2$
c) Return $sqrt(b)$

### 6.2.3 Polynomial generation

This section specifies techniques for generating degree $N$-$1$ polynomials corresponding to particular distributions.

- A uniform polynomial mod $q$ is generated by creating for each polynomial, a list of coefficients within the range of 0 and $q$-$1$, where $q$ is the maximum of the absolute value of each coefficient;
- A fixed weight binary polynomial is generated by creating for each polynomial, a list of length $d$ containing the indices of the +1 coefficients;
- A fixed weight trinary polynomial is generated by creating for each polynomial, a list of length $d1$ containing the indices of the +1 coefficients, followed by a list of length $d2$ containing the indices of the -1 coefficients;
- A random polynomial sampled from discrete Gaussian distribution $\chi_\sigma^N$ with dimension $N$ and standard deviation σ is generated by creating for each polynomial, a list of coefficients that are sampled from $\chi_\sigma$.

Note: The algorithms described below are a reference instantiation of above generators. The implementer MAY choose their own algorithms to generate polynomials as long as they meet the desired property.

#### 6.2.3.1 Uniform mod *q* polynomial generator

A uniform mod $q$ polynomial generator is instantiated with parameters $N$ and $q$, and a string $s$ for randomness. It produces a degree $N$-$1$ polynomial whose coefficients are uniformly randomly distributed between $0$ and $q$-$1$.

**Input:** $N$, $q$, $s$.

**Output:** A pseudo random polynomial *f* all of whose coefficients lie between 0 and *q*-1.

**Operation:** The polynomial shall be computed by the following or an equivalent sequence of steps:

a) Set $f := 0$
b) for (i=0; i<N; i++)
   1) $k$ = pop *(log q +1)* bits from *s*
   2) Set $f_i := k \% q$
c) Return *f*

#### 6.2.3.2 Fixed weight binary polynomial generator

A binary polynomial generator is instantiated with parameter *N* and $d_f$ , and a string *s* for randomness, and produces a binary polynomial with $d_f$ +1s.

**Input:** $N$, $d_f$, $s$.

**Output:** A binary polynomial *f*.

**Operation:** The polynomial shall be computed by the following or an equivalent sequence of steps:

a) Set $f := 0$
b) Set $t := 0$
c) While $t < d_f$ do
   1) $k$ = pop *(log N +1)* bits from *s*
   2) $i = k \% N$
   3) If $f_i = 0$
      i) Set $f_i := 1$
      ii) Set $t := t + 1$
d) Return *f*.

#### 6.2.3.3 Fixed weight trinary polynomial generator

A trinary polynomial generator is instantiated with parameter *N* and *d*, and a string *s* for randomness, and produces a trinary polynomial with $d_1$ +1s and $d_2$ -1s, represented as a series of indices.

**Input:** $N$, $d_1$, $d_2$, $s$.

**Output:** A trinary polynomial *f*.

**Operation:** The polynomial shall be computed by the following or an equivalent sequence of steps:

a) Set $f := 0$
b) Set $t := 0$
c) While $t < d_1$ do
   1) $k$ = pop *(log N +1)* bits from *s*
   2) $i = k \% N$
   3) If $f_i = 0$
      i) Set $f_i := 1$
      ii) Set $t := t + 1$

d) Set $t := 0$
e) While $t < d_2$ do
   1) $k$ = pop *(log N +1)* bits from $s$
   2) $i = k \% N$
   3) If $f_i = 0$
      i) Set $f_i := -1$
      ii) Set $t := t + 1$
f) Return $f$.

#### 6.2.3.4 Discrete Gaussian polynomial generator

A discrete Gaussian polynomial generator is instantiated with parameters $N$, a discrete Gaussian sampler over the integers (iDGS) with a string $s$ for randomness and a standard deviation σ. It produces a degree $N-1$ polynomial that follows discrete Gaussian distribution.

**Input:** $N, s, \sigma$.

**Output:** A $f$ that follows discrete Gaussian distribution.

**Operation:** The polynomial shall be computed by the following or an equivalent sequence of steps:

a) Set $f := 0$
b) for (i=0; i<N; i++)
   1) Call the iDGS with input $s$ to obtain an integer $k$.
   2) Set $f_i := k$
c) Return $f$

##### *6.2.3.4.1 Discrete Gaussian sampler over the integers*

A discrete Gaussian sampler over the integers (iDGS) is instantiated with a string $s$ for randomness and a standard deviation σ. It produces a degree $N$ polynomial that follows discrete Gaussian distribution.

**Input:** $s, \sigma, t$.

**Output:** An integer that follows discrete Gaussian distribution with $t$ bits precision.

**Operation:** The polynomial shall be computed by the following or an equivalent sequence of steps:

a) $a$ = Pop $t$ bits from $s$;
b) $b$ = Pop $t$ bits from $s$;
c) Set $a = a/2^t$, $b = b/2^t$
d) Set $r = \sqrt{-2\log b}\sigma$ and $\theta = 2\pi a$;
e) Set $s_1 = \lfloor r\sin\theta \rceil$ and $s_2 = \lfloor r\cos\theta \rceil$

NOTE: this is the Box-Muller sampler. It indeed generates two Gaussian integers at a time. However, it only provides basic functionalities. It has a limited precision ($t$ bits). One MAY choose higher precision and/or constant-time Gaussian samplers if desired.

## *6.3 Polynomial arithmetics over the integer domain*

Let **Z** be the ring of integers. The polynomial ring over **Z**, denoted **Z**[*X*], is the set of all polynomials with coefficients in the integers.

### 6.3.1 Polynomial multiplication

The *convolution polynomial ring (over* **Z***) of degree N* is the quotient ring of either $\mathbf{Z}[X]/(X^N - 1)$ or $\mathbf{Z}[X]/(X^N + 1)$. All multiplications of polynomials *a* and *b*, represented as $a \times b$, are taken to occur in the ring $\mathbf{Z}[X]/(X^N - 1)$ or $\mathbf{Z}[X]/(X^N + 1)$ unless otherwise noted. Other rings are possible and may be supported in future versions of this standard. An example of those rings is $\mathbf{Z}[X]/(X^N - \mathrm{X} - 1)$. See [B32] for more details.

### 6.3.2 Inversion in a polynomial ring

For certain cryptographic operations such as key generation, it is necessary to take the inverse of a polynomial in the ring. For the parameter sets in this document the ring is either $(\mathbf{Z}/q\mathbf{Z})[X]/(X^N - 1)$ or $(\mathbf{Z}/q\mathbf{Z})[X]/(X^N + 1)$, though in principle it may be a different ring or even a field such as $(\mathbf{Z}/q\mathbf{Z})[X]/(X^N - X - 1)$. 6.4.4.1 and 6.4.4.2 describe the algorithms necessary for inversion in general. 6.4.4.3 and 6.4.4.4 specify an optimization for $(\mathbf{Z}/q\mathbf{Z})[X]/(X^N - 1)$ where *q* is a power of *2*.

#### 6.3.2.1 The Polynomial Division Algorithm in $\mathbf{Z}_p[X]$

This algorithm divides one polynomial by another polynomial in the ring of polynomials with integer coefficients modulo a prime *p*. All convolution operations occur in the ring $\mathbf{Z}_p[X]$ in this algorithm (i.e. there is no modular reduction of the powers of the polynomials).

**Input**: A prime *p*, a polynomial *a* in $\mathbf{Z}_p[X]$ and a polynomial *b* in $\mathbf{Z}_p[X]$ of degree *N*-1 whose leading coefficient $b_N$ is not 0.

**Output**: Polynomials *q* and *r* in $\mathbf{Z}_p[X]$ satisfying $a = b \times q + r$ and $\deg r < \deg b$.

**Operation:** Polynomial Division Algorithm in $\mathrm{Z}_p[X]$ shall be computed by the following or an equivalent sequence of steps;

a) Set $r := a$ and $q := 0$
b) Set $u := b_N^{-1} \bmod p$
c) While $\deg r >= N$ do
   1) Set $d := \deg r(X)$
   2) Set $v := u \times r_d \times X^{(d-N)}$
   3) Set $r := r - v \times b$
   4) Set $q := q + v$
d) Return *q*, *r*

#### 6.3.2.2 The Extended Euclidean Algorithm in $\mathrm{Z}_p[X]$

The Extended Euclidean Algorithm finds a greatest common divisor *d* (there may be more than one that are constant multiples of each other) of two polynomials *a* and *b* in $\mathbf{Z}_p[X]$ and polynomials *u* and *v* such that $a \times u + b \times v = d$. All convolution operations occur in the ring $\mathbf{Z}_p[X]$ in this algorithm (i.e. there is no modular reduction of the powers of the polynomials).

**Input**: A prime *p* and polynomials *a* and *b* in $\mathbf{Z}_p[X]$ with *a* and *b* not both zero.

**Output**: Polynomials *u*, *v*, *d* in $\mathbf{Z}_p[X]$ with $d = \mathrm{GCD}(a, b)$ and $a \times u + b \times v = d$.

**Operation:** Extended Euclidean Algorithm in $Z_p[X]$ shall be computed by the following or an equivalent sequence of steps;

a) If $b = 0$ then return $(1, 0, a)$
b) Set $u := 1$
c) Set $d := a$
d) Set $v_1 := 0$
e) Set $v_3 := b$
f) While $v_3 \neq 0$ do
   1) Use the division algorithm (6.4.4.1) to write $d = v_3 \times q + t_3$ with $\deg t_3 < \deg v_3$
   2) Set $t_1 := u - q \times v_1$
   3) Set $u := v_1$
   4) Set $d := v_3$
   5) Set $v_1 := t_1$
   6) Set $v_3 := t_3$
g) Set $v := (d - a \times u)/b$ [This division is exact, i.e., the remainder is 0]
h) Return $(u, v, d)$

### 6.3.2.3 Inverses in $\mathbf{Z}_p[X]/(X^N - 1)$

The Extended Euclidean Algorithm may be used to find the inverse of a polynomial $a$ in $\mathbf{Z}_p[X]/(X^N - 1)$ if the inverse exists. The condition for the inverse to exist is that $\mathrm{GCD}(a, X^N - 1)$ should be a polynomial of degree 0 (i.e. a constant). All convolution operations occur in the ring $\mathbf{Z}_p[X]/(X^N - 1)$ in this algorithm.

**Input**: A prime $p$, a positive integer $N$ and a polynomial $a$ in $\mathbf{Z}_p[X]/(X^N - 1)$.

**Output**: A polynomial $b$ satisfying $a \times b = 1$ in $\mathbf{Z}_p[X]/(X^N - 1)$ if $a$ is invertible in $\mathbf{Z}_p[X]/(X^N - 1)$, otherwise FALSE.

**Operation:** Inverses in $Z_p[X]/(X^N - 1)$ shall be computed by the following or an equivalent sequence of steps;

a) Run the Extended Euclidean Algorithm (6.4.4.2) with input $a$ and $(X^N - 1)$. Let $(u, v, d)$ be the output, such that $a \times u + (X^N - 1) \times v = d = \mathrm{GCD}(a, (X^N - 1))$.
b) If $\deg d = 0$
c) Return $b = d^{-1} \pmod p \times u$
d) Else return FALSE

### 6.3.2.4 Inverses in $\mathbf{Z}_{p^\wedge r}[X]/(X^N - 1)$

For key generation in this standard it is necessary to calculate inverses in $Z_a[X]/(X^N - 1)$, where $q$ is a power of 2. In this case, the Inversion Algorithm (6.4.4.3) may be used to find the inverse of $a(X)$ in the quotient ring $(R/2R)[X]/(M(X))$. Then the following algorithm may be used to lift it to an inverse of $a(X)$ in the quotient ring $(R/p^eR)[X]/(M(X))$ with higher powers of the prime 2 (or any prime $p$).

**Input**. A prime $p$ in a Euclidean ring $R$, a monic polynomial $M(X)$ □$R[X]$, a polynomial $a(X)$ □ $R[X]$, and an exponent $e$.

**Output**. An inverse $b(X)$ of $a(X)$ in the ring $(R/p^eR)[X]/(M(X))$ if the inverse exists, otherwise FALSE.

a) Use the Inversion Algorithm 6.4.4.4 to compute a polynomial $b(X)$ □ $R[X]$ that gives an inverse of $a(X)$ in $(R/pR)[X]/(M(X))$. Return FALSE if the inverse does not exist. [The Inversion Algorithm may be applied here because $R/pR$ is a field, and so $(R/pR)[X]$ is a Euclidean ring.]

b) Set $n \leftarrow 2$

c) While $e > 0$ do

d) $b(X) \leftarrow 2 \times b(X) - a(X) \times b(X)^2$ (mod $M(X)$), with coefficients computed modulo $p^n$

e) Set e $\leftarrow$ □ $e/2$ □

f) Set $n \leftarrow 2 \times n$

g) Return $b(X)$ mod $M(X)$ with coefficients computed modulo $p^e$.

## *6.4 Polynomial arithmetics over NTT*

For two polynomials in their NTT form, namely, $a = \langle a_0, a_1, \ldots, a_{N-1} \rangle$ and $b = \langle b_0, b_1, \ldots, b_{N-1} \rangle$, their arithmetic operations (+, -, ×, and /) are carried out coefficient-wise. For example the following algorithm computes c = a \$ b for \$ in {+, -, ×, /}.

**Input**: A prime $p$, a positive integer $N$ and polynomials $a = \langle a_0, a_1, \ldots, a_{N-1} \rangle$ and $b = \langle b_0, b_1, \ldots, b_{N-1} \rangle$.

**Output**: A polynomial $c = a \times b$ in its NTT form.

**Operation:** The multiplication shall be computed by the following or an equivalent sequence of steps;

a) for (i=0; i<N; i++)

   1) $c_i = a_i \,\$\, b_i \bmod q$

b) Return $c$

In particular, the inverse of a polynomial $a = \langle a_0, a_1, \ldots, a_{N-1} \rangle$ is simply $\langle 1/a_0, 1/a_1, \ldots, 1/a_{N-1} \rangle \bmod q$.

# 7 Data Types and Conversions

## *7.1 Bit Strings and Octet Strings*

As usual, a **bit** is defined to be an element of the set {0, 1}. A **bit string** is defined to be an ordered array of bits. A **byte** (also called an **octet**) is defined to be a bit string of length 8. A **byte string** (also called an **octet string**) is an ordered array of bytes. The terms **first** and **last**, **leftmost** and **rightmost**, **most significant** and **least significant**, and **leading** and **trailing** are used to distinguish the ends of these sequences (**first**, **leftmost**, **most significant** and **leading** are equivalent; **last**, **rightmost**, **least significant** and **trailing** are equivalent). Within a byte, we additionally refer to the **high-order** and **low-order** bits, where **high-order** is equivalent to **first** and **low-order** is equivalent to **last**.

Note that when a string is represented as a sequence, it may be indexed from left to right or from right to left, starting with any index. For example, consider the octet string of two octets: 2a 1b. This corresponds to the bit string 0010 1010 0001 1011. No matter what indexing system is used, the first octet is still 2a, the first bit is still 0, the last octet is still 1b, and the last bit is still 1. The high-order bit of the second octet is 0; the low-order bit of the second octet is 1.

When a bit string or a octet string is being encoded into a polynomial with coefficients reduced mod $q$ (a "ring element"), where $q$ is usually either 128 or 256, the integer coefficients are mapped individually to bit or octet strings, which are then concatenated. This mapping and its reverse are described in the conversion primitives OS2REP, BS2REP, RE2OSP and RE2BSP from 7.5 to 7.8.

This standard does not specify a single algorithm for converting from bit/octet strings to trinary polynomials in an unbiased and reversible fashion. Instead, the standard uses two algorithms, which are defined inline in the techniques that use them. One algorithm is reversible but biased; the other is unbiased but non-reversible.

## 7.2 Converting Between Integers and Bit Strings (I2BSP and BS2IP)

### 7.2.1 Integer to Bit String Primitive (I2BSP)

I2OSP converts a nonnegative integer to a bit string of a specified length.

**Input:** $i$, nonnegative integer to be converted; $bLen$, intended length of the resulting bit string

**Output:** $B$, corresponding bit string of length $bLen$

**Operation:** The output shall be computed by the following or an equivalent sequence of steps:

a) If $x \geq 2^{xLen}$, output "integer too large" and stop.

b) Write the integer $x$ in its unique $xLen$-bit representation in base 2: $x = x_{xLen-1} \times 2^{xLen-1} + x_{xLen-2} \times 2^{xLen-2} + \ldots + x_1 \times 2 + x_0$ where $x_i = 0$ or 1 (note that one or more leading bits will be zero if $x$ is less than $2^{xLen-1}$).

c) Output the bit string $x_{xLen-1}\ x_{xLen-2} \ldots x_1\ x_0$.

### 7.2.2 Bit String to Integer Primitive (BS2IP)

BS2IP converts a bit string to a nonnegative integer.

**Input:** $B$, bit string to be converted ($bLen$ is used to denote the length of $B$)

**Output:** $x$, corresponding nonnegative integer

**Operation:** The output shall be computed by the following or an equivalent sequence of steps:

a) If $B$ is of length 0, output 0.

b) Let $b_{bLen-1}\ b_{bLen-2} \ldots b_1\ b_0$ be the bits of $B$ from leftmost to rightmost.

c) Let $x = b_{bLen-1} \times 2^{bLen-1} + b_{bLen-2} \times 2^{bLen-2} + \ldots + b_1 \times 2 + b_0$.

d) Output $x$.

## 7.3 Converting Between Integers and Octet Strings (I2OSP and OS2IP)

### 7.3.1 Integer to Octet String Primitive (I2OSP)

I2OSP converts a nonnegative integer to an octet string of a specified length.

**Input:** $x$, nonnegative integer to be converted; $oLen$, intended length of the resulting octet string

**Output:** $O$, corresponding octet string of length $oLen$

**Operation:** The output shall be computed by the following or an equivalent sequence of steps:

a) If $x \geq 256^{oLen}$, output "integer too large" and stop.

b) Write the integer $x$ in its unique $oLen$-digit representation in base 256: $x = o_{oLen-1} \times 256^{oLen-1} + o_{oLen-2} \times 256^{oLen-2} + \ldots + o_1 \times 256 + o_0$

where $0 \leq o_i < 256$ (note that one or more leading digits will be zero if $o$ is less than $256^{oLen-1}$).

c) For for $1 \leq x \leq oLen$, let the octet $O_i$ be the concatenation of the bits in the integer representation of $o_{oLen-i}$ , where left-most bit of the octet is the high order bit of the binary representation. Output the octet string

$O = O_1\ O_2 \ldots O_{oLen}$.

NOTE—As an example, the integer 944 has the three-digit representation $944 = 0 \times 256^2 + 3 \times 256 + 178$. The corresponding octet string, expressed in integer values, is 0 3 178; as binary values, it is

00000000 00000011 10110010

and in hexadecimal it is 00 03 b2.

### 7.3.2 Octet String to Integer Primitive (OS2IP)

OS2IP converts an octet string to a nonnegative integer.

**Input:** $x$, nonnegative integer to be converted; $oLen$, intended length of the resulting octet string

**Output:** $O$, corresponding octet string of length $oLen$

**Operation:** The output shall be computed by the following or an equivalent sequence of steps:

a) If $O$ is of length 0, output 0.

b) Let $O_1\ O_2 \ldots O_{oLen}$ be the octets of $O$ from first to last, and let $o_{oLen-j}$ be the integer value of the octet $O_j$ for $1 \leq j \leq oLen$, where the integer value is represented as an octet (x.e., an eight-bit string) most significant bit first.

c) Output $x = o_{oLen-1} \times 256^{oLen-1} + o_{oLen-2} \times 256^{oLen-2} + \ldots + o_1 \times 256 + o_0$.

## 7.4 Converting Between Bit Strings and Right-Padded Octet Strings (BS2ROSP and ROS2BSP)

This clause gives the primitives used to convert between bit strings and right-padded octet strings.

### 7.4.1 Bit String to Right-Padded Octet String Primitive (BS2ROSP)

**Input:** $B$: bit string to be converted; $oLen$: intended length of the resulting octet string

**Output:** $O$, corresponding octet string of length $oLen$

**Operation:** The output shall be computed by the following or an equivalent sequence of steps:

a) Set $bLen$ equal to the length of $x$ in bits.

b) If $bLen > 8 \times oLen$, output “input too long” and stop.

c) Append $(8 \times oLen - bLen)$ zero bits to the end of $x$.

d) Let $b_0\ b_1 \ldots b_{xLen-2}\ b_{xLen-1}$ be the bits of $B$ from first to last. For $0 \leq i < oLen - 1$, let the octet $O_i = b_{8i}\ b_{8i+1} \ldots b_{8i+7}$. Output the octet string

$O = O_0\ O_1 \ldots O_{oLen-1}$.

### 7.4.2 Right-Padded Octet String to Bit String Primitive (ROS2BSP)

ROS2BSP converts an octet string to a bit string of a specified length.

**Input:** *O*: octet string to be converted; *bLen*: intended length of the resulting bit string

**Output:** *B*: corresponding bit string of length *bLen*

**Operation:** The output shall be computed by the following or an equivalent sequence of steps:

a) Set *oLen* equal to the length of *O* in octets.
b) If $bLen > 8 \times oLen$, output "input too short" and stop.
c) For $0 \le i < oLen - 1$, consider the octet $O_i$ to be the bits $b_{8i}\ b_{8i+1} \ldots b_{8i+7}$.
d) If any of the bits $b_{bLen\ -1} \ldots b_{8 \times oLen\ -1}$ are non-zero, output "non-zero bits found after end of bit string" and stop.
e) Output the bit string
$B = b_0\ b_1 \ldots b_{bLen-1}$.

## 7.5 Converting Between Ring Elements and Bit Strings (RE2BSP and BS2REP)

While octet string representation may be most convenient for ring element arithmetic in a microprocessor, ring elements may be more compactly stored and transmitted as bit strings. This clause provides the appropriate conversion primitives.

### 7.5.1 Ring Element to Bit String Primitive (RE2BSP)

RE2BSP converts a ring element to a bit string.

**Input:** *a*: ring element to be converted, equal to $a_0 + a_1 X + a_2 X^2 + \ldots + a_{N-1} X^{N-1}$; *N*: dimension of ring; *q*: larger modulus: all coefficients of the ring element are between 0 and *q*-1.

**Output:** *B*: resulting bit string.

**Operation:** The output shall be computed by the following or an equivalent sequence of steps:

a) For $j = 0$ to $N$-1:
b) Set $A_j$ equal to the smallest positive representation of $a_j$ mod $q$.
c) Set $B_j$ = I2BSP ($A_j$, ceil[$\log_2 q$]). If any of the calls to I2BSP output an error, output that error and stop.
d) Output the bit string $B = B_0\ B_1 \ldots B_{N-1}$.

NOTE—As an example, if $q$=128 and $N$=5, the polynomial

$a[X] = 45 + 2X + 77\ X^2 + 103\ X^3 + 12\ X^4$

is represented by the bit string 0101101 0000010 1001101 1100111 0001010. (If this were subsequently to be converted to an octet string using BS2ROSP, it would become first the bit string 0101 1010 0000 1010 0110 1110 0111 0001 0100 0000, and then the octet string 5a 0a 6e 71 40).

### 7.5.2 Bit String to Ring Element Primitive (BS2REP)

BS2REP converts a bit string to a ring element.

**Input:** *B*: bit string to be converted; *N*: dimension of ring; *q*: larger modulus: all coefficients of the ring element are between 0 and *q*-1.

**Output:** *a*: resulting ring element, equal to $a_0 + a_1 X + a_2 X^2 + \ldots + a_{N-1} X^{N-1}$

**Operation:** The output shall be computed by the following or an equivalent sequence of steps:

a) If the length of $B$ is not equal to $N \times$ ceil[$\log_2 q$], output “bit string incorrect length” and stop.
b) Consider $B$ to be the series of bit strings $B = B_0 B_1 \ldots B_{N-1}$., where each $B_j$ is of length ceil[$\log_2 q$] bits.
c) For $j = 0$ to $N$-1, set $a_j$ = BS2IP ($B_j$). If BS2IP outputs an error, output “error”.
d) Output $a = a_0 + a_1 X + a_2 X^2 + \ldots + a_{N-1} X^{N-1}$.

## 7.6 Converting Between Ring Elements and (Compact) Octet Strings (RE2OSP and OS2REP)

This clause gives the primitives for converting between ring elements and octet strings using the minimum number of bits per coefficient.

### 7.6.1 Ring Element to Octet String Primitive (RE2OSP)

RE2OSP converts a ring element to an octet string.

**Input:** $a$: ring element to be converted, equal to $a_0 + a_1 X + a_2 X^2 + \ldots + a_{N-1} X^{N-1}$; $N$: dimension of ring; $q$: larger modulus to be passed to RE2BSP: all coefficients of the ring element are between 0 and $q$-1

**Output:** $O$: corresponding octet string

**Operation:** The output shall be computed by the following or an equivalent sequence of steps:

a) Convert the ring element $a$ to a bit string $bA$ using RE2BSP.
b) Convert the bit string $bA$ to the octet string $O$ using BS2ROSP.
c) Output O.

### 7.6.2 Octet String to Ring Element Primitive (OS2REP)

OS2REP converts an octet string to a ring element.

**Input:** $O$: octet string to be converted; $N$: dimension of ring; $q$: larger modulus: all coefficients of the ring element are between 0 and $q$-1.

**Output:** $a$: resulting ring element, equal to $a_0 + a_1 X + a_2 X^2 + \ldots + a_{N-1} X^{N-1}$

**Operation:** The output shall be computed by the following or an equivalent sequence of steps:

a) If the length of $O$ is not equal to $N \times$ ceil[$\log_{256} q$], output “octet string incorrect length” and stop.
b) Convert the octet sting $O$ to the bit string $bA$ using ROS2BSP.
c) Convert the bit string $bA$ to the ring element $a$ using BS2REP.
d) Output $a$.

## 7.7 Converting Between Ring Elements and Padded Octet Strings (RE2POSP and POS2REP)

This clause gives the primitives for converting between ring elements and octet strings such that coefficients of the ring element do not cross octet boundaries.

### 7.7.1 Ring Element to Padded Octet String Primitive (RE2POSP)

RE2OSP converts a ring element to an octet string.

**Input:** *a*: ring element to be converted, equal to $a_0 + a_1 X + a_2 X^2 + \ldots + a_{N-1} X^{N-1}$; *N*: dimension of ring; *q*: larger modulus to be passed to RE2BSP: all coefficients of the ring element are between 0 and *q*-1

**Output:** *O*: corresponding octet string

**Operation:** The output shall be computed by the following or an equivalent sequence of steps:

a) For $j = 0$ to $N$-1:
b) Set $A_j$ equal to the smallest positive representation of $a_j$ mod $q$.
c) Set $O_j$ = I2OSP ($A_j$, ceil[$\log_{256} q$]). If any of the calls to I2OSP output an error, output that error and stop.
d) Output the octet string $O = O_0 O_1 \ldots O_{N-1}$.

### 7.7.2 Octet String to Ring Element Primitive (OS2REP)

OS2REP converts an octet string to a ring element.

**Input:** *O*: octet string to be converted; *N*: dimension of ring; *q*: larger modulus: all coefficients of the ring element are between 0 and *q*-1.

**Output:** *a*: resulting ring element, equal to $a_0 + a_1 X + a_2 X^2 + \ldots + a_{N-1} X^{N-1}$

**Operation:** The output shall be computed by the following or an equivalent sequence of steps:

a) If the length of *O* is not equal to $N \times$ ceil[$\log_{256} q$], output "octet string incorrect length" and stop.
b) Consider *O* to be the series of octet strings $O = O_0 O_1 \ldots O_{N-1}$., where each $O_j$ is of length ceil[$\log_{256} q$] bits.
c) For $j = 0$ to $N$-1, set $a_j$ = OS2IP ($O_j$). If OS2IP outputs an error, output "error".
d) Output $a = a_0 + a_1 X + a_2 X^2 + \ldots + a_{N-1} X^{N-1}$.

## *7.8 Converting Between Trinary Ring Elements and Octet Strings (TRE2OSP and OS2TREP)*

This clause gives the primitives for converting between ring elements and octet strings such that coefficients of the ring element do not cross octet boundaries.

### 7.8.1 Trinary Ring Element to Octet String Primitive (TRE2OSP)

RE2OSP converts a trinary ring element to an octet string.

**Input:** *a*: ring element to be converted, equal to $a_0 + a_1 X + a_2 X^2 + \ldots + a_{N-1} X^{N-1}$, where the coefficients $a_i$ are all in the set {-1, 0, 1}; *N*: dimension of ring

**Output:** *O*: corresponding octet string

**Operation:** The output shall be computed by the following or an equivalent sequence of steps:

a) Set $k = 0$
b) For $j = 0$ to $N$-1:
   1) If $a_j = 1$
      i) Set $O_k$ = I2OSP (j, ceil[$\log_{256} N$])
      ii) Set $k = k+1$

c) For $j = 0$ to $N$-1:
   1) If $a_j = -1$
      i) Set $O_k$ = I2OSP (j, ceil[$\log_{256} N$])
      ii) Set $k = k$+1
d) Output the octet string $O = O_0\ O_1 \ldots O_{N-1}$.

### 7.8.2 Octet String to Trinary Ring Element Primitive (OS2TREP)

OS2REP converts an octet string to a ring element whose coefficients are all in the set {-1, 0, 1}.

**Input:** $O$: octet string to be converted; $N$: dimension of ring; $d_+$, expected number of +1s; $d_-$, expected number of -1s.

**Output:** $a$: resulting ring element, equal to $a_0 + a_1 X + a_2 X^2 + \ldots + a_{N-1} X^{N-1}$

**Operation:** The output shall be computed by the following or an equivalent sequence of steps:

a) If the length of $O$ is not equal to $(d_+ + d_-) \times$ ceil[$\log_{256} N$], output "octet string incorrect length" and stop.
b) Consider $O$ to be the series of octet strings $O = O_0\ O_1 \ldots O_{(d+\ +\ d-)}$., where each $O_j$ is of length ceil[$\log_{256} N$] bits.
c) For $k = 0$ to $d_+$:
   1) If k != 0
      i) If OS2IP ($O_k$) <= OS2IP ($O_{k-1}$), output "error".
   2) Set $a_{\mathrm{OS2IP}\,(Ok)} = 1$. If OS2IP outputs an error, output "error".
d) For $k = d_+$+1 to $d_+ + d_-$:
   1) If k != $d_+$+1
      i) If OS2IP ($O_k$) <= OS2IP ($O_{k-1}$), output "error".
   2) Set $a_{\mathrm{OS2IP}\,(Ok)} = 1$. If OS2IP outputs an error, output "error".
e) Output $a = a_0 + a_1 X + a_2 X^2 + \ldots + a_{N-1} X^{N-1}$.

# 8 Supporting algorithms

## *8.1 Overview*

In order to perform the operations securely, implementers shall choose supporting algorithms that satisfy the security needs of the schemes. The security level of the supporting algorithm typically depends on the desired security level of the scheme (e.g. for a desired security level of 80 bits, the SHA-1 hash algorithm described in FIPS 180 is typically chosen). This clause defines the algorithms that shall be used to meet this standard.

## *8.2 Hash Functions and XOFs*

Hash functions are essential to generate blinding polynomials in the encryption scheme and the signature scheme. For security purposes, the hash function should be chosen at a strength commensurate to the desired security level. We require the hash function to be collision resistant and preimage resistant.

The recommended hash functions are SHA512 from SHA2 family and SHA3-512 from SHA3 (Keccak) family (see FIPS 180 [B7]). Although, to allow customized input of initial vectors, the recommended parameter sets in this document does not specify hash functions.

All hash functions in this standard take an octet string as an input and produce an octet string as an output. For compatibility with other standards which specify input and output as bit strings, the conversion primitives ROS2BSP and BS2ROSP (clauses 7.4.1 and 7.4.2) may be used.

# 9 NTRU-KEM

The following clause defines the supported public key key encapsulation schemes.

## 9.1 KEM Scheme Operations

The NTRU-KEM scheme consists of the five operations key generation, key pair validation, public key validation, encryption and decryption. These operations are defined generally in this clause as operations parameterized by Components, without assuming any specific choices of the Components. Specific choices of Components to meet specific security goals are specified in the Parameters section, 9.2.

### 9.1.1 Key Generation

A key pair shall be generated using the following or a mathematically equivalent set of steps. Note that the algorithm below outputs only the values $f$ and $h$. In some applications it may be desirable to store the values $f^{-1}$ and $g$ as well. This standard does not specify the output format for the key as long as it is unambiguous.

**Components:** The parameters $N$, $q$, $p$, $dF$, $d_g$.

**Input**: A string $s$ as a the source of randomness.

**Output:** A key pair consisting of the private key $F$ and the public key $h$

**Operation:** The key pair shall be computed by the following or an equivalent sequence of steps:

a) Set the polynomial $F := 0$.
b) Call the fix weight trinary polynomial generator of section 6.4.1.2 with input $N$, $dF+1$, $dF$ and s to obtain a trinary polynomial $F$.
c) Compute the polynomial $f := 1 + p \times F$
d) Compute the polynomial $f^{-1}$ (i.e. the polynomial $f^{-1}$ such that $f^{-1} \times f = f \times f^{-1} = 1$) in the ring. If $f^{-1}$ does not exist, go to step a).
e) Call the fix weight trinary polynomial generator of section 6.4.1.2 with input $N$, $d_g$ and s to obtain a trinary polynomial $g$.
f) Compute the polynomial $h := f^{-1} \times g \times p$ in the ring
g) Output $F$, $h$

### 9.1.2 Encapsulation operations

This clause defines the encapsulation operations. Note: the encapsulation operation simply encapsulates a ring element. It does not check for the validity of this element. The implementor needs to ensure there is enough entropy in this element at the protocol level.

**Components**:

- The length of the input message $m$, *lLen* in bytes.
- The fixed weight trinary polynomial generation function.
- The length of the encoding buffer, *bufferLenBits*

**Inputs**:

- The message *m*, which is a trinary polynomial
- The public key *h*
- A string *s* as a the source of randomness.

**Output**: The ciphertext *e*, which is a ring element

**Operation**: The ciphertext *e* shall be calculated by the following or an equivalent sequence of steps:

a) Use the fixed weight trinary polynomial generation function with the seed *s* and parameters N, dr to produce *r*.
b) Calculate $R = r \times h \bmod q$.
c) Calculate the ciphertext as e = R + m mod *q*.
d) Output e.

### 9.1.3 Decryption Operation

This clause defines the decryption operation.

**Components**:

- None.

**Inputs**:

- The ciphertext *e*, which is a polynomial of degree *N*-1.
- The private key *F*.
- The public key *h*.

**Output**: An element from the polynomial ring.

**Operation**: The message m shall be calculated by the following or an equivalent sequence of steps:

a) Compute $f = pF+1$
b) Compute $cm = f*e \bmod p$
c) Output *cm* as the decrypted message *m*.

### 9.1.4 Key Pair Validation Methods

A key pair validation method determines whether a candidate public-key/private-key pair meets the constraints for key pairs produced by a particular key generation method.

#### 9.1.4.1 Key Pair Validation for Trinary Keys

This key validation method corresponds to the key generation operation in 9.1.1.

**Components:** The parameters $N$, $q$, $dF$, $d_g$,

**Input:** The private key component *F* and the public key *h*.

**Output:** “valid” or “invalid”.

**Operation:**

a) Check that *h* is a polynomials of degree no greater than *N*-1. If not, output “invalid” and stop.

b) Check that all of the coefficients of $h$ lie in the range $[0, q\text{-}1]$. If any coefficients lie outside this range, output "invalid" and stop.

c) Convert *private_key_blob* into a polynomial $F$.

d) Check that $F$ is trinary with exactly $dF+1$ 1s and $dF$ -1s. If not, output "invalid" and stop.

e) Check that $F$ is a polynomial of degree no greater than $N$-1. If not, output "invalid" and stop.

f) Set $f = 1 + 3F \bmod q$.

g) Set $g = f \times h \bmod q$.

h) Check that $g$ is trinary with exactly $(d_g +1)$ 1s and $d_g$ -1s. If it is not, output "invalid" and stop.

i) Output "valid".

## 9.1.5 Public-key validation

### 9.1.5.1 Full public-key validation

A full public-key validation method determines whether a candidate public key satisfies the definition of a public key and meets any additional constraints imposed by a given key pair generator. Such methods provide the highest assurance to a relying party. For example, for keys generated using the key generation operation in 9.1.1, full public-key validation would prove that $h = f^{-1}g \bmod q$, where $f = 1+pF$ and $F$, $g$ have $dF$, $d_g$ 1s respectively. Currently there are no known methods that provide full public-key validation for the above schemes in this standard.

### 9.1.5.2 Partial public-key validation and plausibility tests

#### *9.1.5.2.1 Overview*

A partial public-key validation method determines, with some level of assurance, whether a candidate public key meets *some* of the properties of a public key. As with full public-key validation methods, partial public-key validation methods may be interactive or non-interactive. This standard supports only non-interactive methods.

Non-interactive methods for LBP-PKE public keys that do not require a witness are called *plausibility tests*. The name reflects the fact that while examining only the public key, the tests only determine whether the public key is plausible, not necessarily whether it is valid. Plausibility tests can detect unintentional errors with reasonable probability, though not with certainty. (See Note.)

This is still an active research area; further methods may be described in future versions of this Standard.

NOTE—There are other ways to detect unintentional errors; a checksum on the key can be used to detect storage and transmission errors, and the signature on a certificate will likely fail verification if the public key is modified. The checks in this clause provide an additional level of assurance beyond the other methods, or an alternative when they are not available.

#### *9.1.5.2.2 Example suite of plausibility tests*

The following is an example of a plausibility test, corresponding to the key generation operation in 9.1.1.

a) Check that $h(1) = g(1)/(1 + pF(1)) \bmod q$. ($F(1) = 0$, $g(1) = 1$ so $h(1) = 1$). If it is not, output "invalid" and stop.

b) For $t = 0$ to $q$-1:

   1) Reduce $h$ into the range $[t, t+q\text{-}1]$.

c) Calculate the centered norm $||h||$ for $h$ reduced into this range.

d) Set $||h||_{\min}$ equal to the minimum value of $||h||$ obtained in the previous step.

e) Set $||r|| = \sqrt{[2\, d_r]}$.

f) If $||h||_{min} > q\ (\sqrt{N})\ /\ (3\ ||r||)$, output “plausible public key” and stop. Otherwise, output “invalid” and stop.

Steps b) to d) are motivated by the observation that for a valid public key $h$, the calculation of $h \times r$ mod $q$ involves a large number of reductions mod $q$. The test checks that $||h \times r|| > q\sqrt{(N)}/2$, in other words that the centered norm of $h \times r$ is with high likelihood greater than the centered norm of a polynomial consisting of $N/2$ coefficients with the value $q/2$ and $N/2$ coefficients with the value $-q/2$ (this calculation uses the pseudo-multiplicative property of the centered norm). For genuine $h$, the typical value of $||h||_{min}$ is slightly under $q\ \sqrt{(N/12)}$. For binary polynomials, the centered norm $||r||$ is $\sqrt{(2d_r)}$, which is considerably greater than $\sqrt{(3)}$ for all parameter sets in this standard. A valid $h$ therefore passes this test with high probability.

## 9.2 Proposed Parameter Sets

This section defines two sets of parameters for NTRU-KEM that give a 128 level of security according to the metrics in this standard.

| Parameter set | p | q | N | max msg length | df | dg | pk len | sk len |
|---|---|---|---|---|---|---|---|---|
| NTRU-KEM-443 | 3 | 2048 | 443 | 55 | 115 | 115 | 610 | 89 |
| NTRU-KEM-743 | 3 | 2048 | 743 | 92 | 247 | 149 | 1022 | 149 |

# 10 NTRU-CCA

The following clause defines the supported public key encryption schemes. In cryptography, one can also achieve CCA-2 security from a CPA secure algorithm via the FO transformation. This transformation usually results into an increase of the ciphertext. Here we use the scheme from \cite{} that achieves CCA-2 security via the NAEP transformation.

## 10.1 Encryption Scheme Operations

The NTRU-CCA encryption scheme consists of the five operations key generation, key pair validation, public key validation, encryption and decryption. These operations are defined generally in this clause as operations parameterized by Components, without assuming any specific choices of the Components. Specific choices of Components to meet specific security goals are specified in the Parameters section, 10.2.

### 10.1.1 Key Generation

The same as the key generation operations in NTRU-KEM.

### 10.1.2 Encryption Operation

This clause defines the Encryption operation.

**Components**:

- The length of the input message *m*, *lLen* in bytes.
- The length of the salt *b*, *sLen* in bytes.
- The fixed weight trinary polynomial generation function.
- The number of bits of public key to hash, *pkLen*.
- The minimum message representative weight, $d_{m0}$.
- The maximum message length *maxMsgLenBytes*.
- The length of the encoding buffer, *bufferLenBits*

**Inputs**:

- The message *m*, which is an octet string of length *l* octets
- The public key *h*

**Output**: The ciphertext *e*, which is a ring element, or "message too long"

**Operation**: The ciphertext *e* shall be calculated by the following or an equivalent sequence of steps:

a) If *lLen* > *maxLen*, output "message too long" and stop.
b) Form the octet string p0, consisting of the 0 byte repeated (*maxMsgLenBytes* + 1 - *lLen*) times.
c) Form the octet string *M* of length *bufferLenBits*/8 as *m* ||*p0*||*b*|| *octL* .
d) Convert *M* to a bit string *Mbin* using OS2BSP. Parse it as a degree *8lLen* polynomial.
e) Pop a *sLen* length of string from *s* as the salt *b*. Parse *b* as a degree *8sLen* polynomial.
f) Compute $m = Mbin + b * X^{N-8sLen-1}$
g) Convert the public key *h* to a bit string *bh* using RE2BSP (7.5.1). Form the bit string *bhTrunc* by taking the first *pkLen* bits of *bh*. Convert *bhTrunc* to the octet string *hTrunc*, of length *pkLen*/8 using BS2OSP. Form *sData* as the octet string *m* || *b* || *hTrunc*
h) Use the fixed weight trinary polynomial generation function with the seed *sData* and parameters N, dr to produce *r*.
i) Calculate $R = r \times h \bmod q$.
j) Use the fixed weight trinary polynomial generation function with the seed *R* and the chosen parameters to produce a polynomial *mask*.
k) Form m' by polynomial addition of *m* and *mask* mod p.
l) If the number of 1s, or -1s, or 0s in m' is less than $d_{m0}$, discard m' and return to step b).
m) Calculate the ciphertext as e = R + m' mod *q*.
n) Output e.

### 10.1.3 Decryption Operation

This clause defines the decryption operation.

**Components**:

- The length of the input ciphertext *c*, *cLen* in bytes.
- The length of the salt *b*, *sLen* in bytes.
- The fixed weight polynomial generation function
- The number of bits of public key to hash, *pkLen*.
- The lower bound *A*
- The minimum message representative weight $d_{m0}$
- The maximum message length *maxMsgLenBytes*

**Inputs**:

-  The ciphertext *e*, which is a polynomial of degree *N*-1.
-  The private key *F* .
-  The public key *h*

**Output**: The message *m*, which is an octet string, or "fail".

**Operation**: The message m shall be calculated by the following or an equivalent sequence of steps:

d) Calculate:
   1) *nLen* = ceil [*N*/8], the number of octets required to hold *N* bits.
   2) *bLen* = *db*/8, the length in octets of the random data
   3) *maxLen* = *nLen* - 1 - *lLen* - *bLen*, the maximum message length.

e) Compute *f* = *pF+1*

f) Decrypt the ciphertext *e* using the selected NTRU decryption primitive with inputs *e* and *f* to get the candidate decrypted polynomial *cm'*.

g) If the number of 1s, or -1s, or 0s in *ci* is less than $d_{m0}$, set "fail" to 1.

h) Calculate the candidate value for $r \times h$, $cR = e - cm' \bmod q$.

o) Use the fixed weight trinary polynomial generation function with the seed *cR* and the chosen parameters to produce a polynomial *mask*

i) Form *cM* by polynomial subtraction of *cm'* and *mask* mod p.

j) Parse *cM* as follows.
   1) The first *lLen* octets represent the message length. Convert the value stored in these octets to the candidate message length *cl*. If *cl* > *maxMsgLenBytes*, set *fail* = 1 and set *cl* = *maxL*.
   2) The next *cl* octets are the candidate message *cm*. the remaining octets should be 0. If they are not, set fail = 1.
   3) The last *bLen* octets are the candidate salt *cb*.

k) Convert the public key *h* to a bit string *bh* using RE2BSP (7.5.1). Form the bit string *bhTrunc* by taking the first *pkLen* bits of *bh*. Convert *bhTrunc* to the octet string *hTrunc*, of length *pkLen*/8 using BS2OSP. Form *sData* as the octet string *cm* || *b* || *hTrunc*

l) Use the fixed weight trinary polynomial generation function with the seed *sData* and parameters N, dr to produce *cr*.

m) Calculate $cR' = h \times cr \bmod q$.

n) If *cR*' != *cR*, set *fail* = 1

o) If *fail* = 1, output "fail". Otherwise, output *cm* as the decrypted message *m*.

### 10.1.4 Key Pair Validation Methods

Same as NTRU-KEM key pair validation methods in section 9.1.4.

### 10.1.5 Public-key validation

Same as NTRU-KEM public key pair validation methods in section 9.1.5.

## *10.2 Possible Parameter Sets*

| Parameter set | p | q | N | max msg length | df | dg | pk len | sk len |
|---|---|---|---|---|---|---|---|---|
| NTRU-KEM-443 | 3 | 2048 | 443 | 34 | 115 | 115 | 610 | 89 |
| NTRU-KEM-743 | 3 | 2048 | 743 | 74 | 247 | 149 | 1022 | 149 |

# 11 SS-NTRU-KEM

The following clause defines the supported public key key encapsulation schemes.

## 11.1 SS-KEM Scheme Operations

The SS-NTRU-KEM scheme consists of the five operations: key generation, key pair validation, public key validation, encryption and decryption. These operations are defined generally in this clause as operations parameterized by Components, without assuming any specific choices of the Components. Specific choices of Components to meet specific security goals are specified in the Parameters section, 11.2.

### 11.1.1 Key Generation

A key pair shall be generated using the following or a mathematically equivalent set of steps. Note that the algorithm below outputs only the values $f$ and $h$. In some applications it may be desirable to store the values $f^{-1}$ and $g$ as well. This standard does not specify the output format for the key as long as it is unambiguous.

**Components:** The parameters $N$, $q$, $p$, *sigma*.

**Input**: A string $s$ as a the source of randomness.

**Output:** A key pair consisting of the private key $F$ and the public key $h$

**Operation:** The key pair shall be computed by the following or an equivalent sequence of steps:

a) Set the polynomial $F := 0$.
b) Call the discrete Gaussian polynomial generator of section 6.4.1.2 with input $N$, *sigma* and s to obtain a Gaussian distributed polynomial $F$.
c) Compute the polynomial $f := 1 + p \times F$
d) Compute the polynomial $f^{-1}$ (i.e. the polynomial $f^{-1}$ such that $f^{-1} \times f = f \times f^{-1} = 1$) in the ring. If $f^{-1}$ does not exist, go to step a).
e) Call the discrete Gaussian polynomial generator of section 6.4.1.2 with input $N$, *sigma* and s to obtain a Gaussian distributed polynomial $g$.
f) Compute the polynomial $h := f^{-1} \times g \times p$ in the ring
g) Output $F$, $h$

### 11.1.2 Encapsulation operations

This clause defines the encapsulation operations. Note: the encapsulation operation simply encapsulates a ring element. It does not check for the validity of this element. The implementor needs to ensure there is enough entropy in this element at the protocol level.

**Components**:

-  The length of the input message *m*, *lLen* in bytes.
-  The discrete Gaussian trinary polynomial generation function.
-  The length of the encoding buffer, *bufferLenBits*

**Inputs**:

-  The message *m*, which is a polynomial mod *p*
-  The public key *h*
-  A string *s* as a the source of randomness.

**Output**: The ciphertext *c*, which is a ring element

**Operation**: The ciphertext *e* shall be calculated by the following or an equivalent sequence of steps:

a) Call the discrete Gaussian polynomial generator with input *N*, *sigma* and s to obtain a Gaussian distributed polynomial *r*.
b) Call the discrete Gaussian polynomial generator of with input *N*, *sigma* and s to obtain a Gaussian distributed polynomial *e*.
c) Calculate $R = r \times h \bmod q$.
d) Calculate the ciphertext as $c = R + p*e + m \bmod q$.
e) Output *c*.

### 11.1.3 Decryption Operation

This clause defines the decryption operation.

**Components**:

-  None.

**Inputs**:

-  The ciphertext *c*, which is a polynomial of degree *N*-1.
-  The private key *F*.
-  The public key *h*.

**Output**: An element from the polynomial ring.

**Operation**: The message m shall be calculated by the following or an equivalent sequence of steps:

a) Compute $f = pF+1$
b) Compute $cm = f*e \bmod p$

### 11.1.4 Key Pair Validation Methods

A key pair validation method determines whether a candidate public-key/private-key pair meets the constraints for key pairs produced by a particular key generation method.

#### 11.1.4.1 Key Pair Validation for Gaussian Keys

This key validation method corresponds to the key generation operation in 11.1.1.

**Components:** The parameters $N$, $q$, *sigma*

**Input:** The private key component $F$ and the public key $h$.

**Output:** "valid" or "invalid".

**Operation:**

a) Check that $h$ is a polynomials of degree no greater than $N$-1. If not, output "invalid" and stop.
b) Check that all of the coefficients of $h$ lie in the range [0, $q$-1]. If any coefficients lie outside this range, output "invalid" and stop.
c) Convert *private_key_blob* into a polynomial $F$.
d) Check that |$F$|_*infty* is smaller than 14*sigma. If not, output "invalid" and stop.
e) Check that $F$ is a polynomial of degree no greater than $N$-1. If not, output "invalid" and stop.
f) Set $f = 1 + 3F \bmod q$.
g) Set $g = f \times h \bmod q$.
h) Check that |$g$|_*infty* is smaller than 14*sigma. If it is not, output "invalid" and stop.
i) Output "valid".

### 11.1.5 Public-key validation

#### 11.1.5.1 Full public-key validation

A full public-key validation method determines whether a candidate public key satisfies the definition of a public key and meets any additional constraints imposed by a given key pair generator. Such methods provide the highest assurance to a relying party. For example, for keys generated using the key generation operation in 9.1.1, full public-key validation would prove that $h = f^{1}g \bmod q$, where $f = 1+pF$ and $F$, $g$ have $dF$, $d_g$ 1s respectively. Currently there are no known methods that provide full public-key validation for the above schemes in this standard.

#### 11.1.5.2 Partial public-key validation and plausibility tests

##### *11.1.5.2.1 Overview*

A partial public-key validation method determines, with some level of assurance, whether a candidate public key meets *some* of the properties of a public key. As with full public-key validation methods, partial public-key validation methods may be interactive or non-interactive. This standard supports only non-interactive methods.

Non-interactive methods for LBP-PKE public keys that do not require a witness are called *plausibility tests*. The name reflects the fact that while examining only the public key, the tests only determine whether the public key is plausible, not necessarily whether it is valid. Plausibility tests can detect unintentional errors with reasonable probability, though not with certainty. (See Note.)

This is still an active research area; further methods may be described in future versions of this Standard.

NOTE—There are other ways to detect unintentional errors; a checksum on the key can be used to detect storage and transmission errors, and the signature on a certificate will likely fail verification if the public key is modified. The checks in this clause provide an additional level of assurance beyond the other methods, or an alternative when they are not available.

#### 11.1.5.2.2 Example suite of plausibility tests

The following is an example of a plausibility test, corresponding to the key generation operation in 9.1.1.

a) Check that $h(1) = g(1)/(1 + pF(1)) \bmod q$. ($F(1) = 0$, $g(1) = 1$ so $h(1) = 1$). If it is not, output “invalid” and stop.

b) For $t = 0$ to $q$-1:

   1) Reduce $h$ into the range $[t, t+q-1]$.

c) Calculate the centered norm $||h||$ for $h$ reduced into this range.

d) Set $||h||_{min}$ equal to the minimum value of $||h||$ obtained in the previous step.

e) Set $||r|| = \surd\ [2\ d_r]$.

f) If $||h||_{min} > q\ (\surd N)\ /\ (3\ ||r||)$, output “plausible public key” and stop. Otherwise, output “invalid” and stop.

Steps b) to d) are motivated by the observation that for a valid public key $h$, the calculation of $h \times r \bmod q$ involves a large number of reductions mod $q$. The test checks that $||h \times r|| > q\surd(N)/2$, in other words that the centered norm of $h \times r$ is with high likelihood greater than the centered norm of a polynomial consisting of $N/2$ coefficients with the value $q/2$ and $N/2$ coefficients with the value $-q/2$ (this calculation uses the pseudo-multiplicative property of the centered norm). For genuine $h$, the typical value of $||h||_{min}$ is slightly under $q\ \surd(N/12)$. For binary polynomials, the centered norm $||r||$ is $\surd(2d_r)$, which is considerably greater than $\surd(3)$ for all parameter sets in this standard. A valid $h$ therefore passes this test with high probability.

## 11.2 Proposed Parameter Sets

| Parameter set | p | q | N | max msg length | sigma | pk len | sk len |
|---|---|---|---|---|---|---|---|
| SS-NTRU-KEM-1024 | 2 | 1073750017 | 1024 | 128 | 724 | 4097 | 4097 |

# 12 SS-NTRU-CCA

The following clause defines the supported public key encryption schemes. This scheme achieves CCA-2 security via the NAEP transformation.

## 12.1 Encryption Scheme Operations

The NTRU-CCA encryption scheme consists of the five operations key generation, key pair validation, public key validation, encryption and decryption. These operations are defined generally in this clause as operations parameterized by Components, without assuming any specific choices of the Components. Specific choices of Components to meet specific security goals are specified in the Parameters section, 11.2.

### 12.1.1 Key Generation

The same as the key generation operations in SS-NTRU-KEM.

### 12.1.2 Encryption Operation

This clause defines the Encryption operation.

**Components**:

- The length of the input message *m*, *lLen* in bytes.
- The length of the salt *b*, *sLen* in bytes.
- The discrete Gaussian polynomial generation function.
- The number of bits of public key to hash, *pkLen*.
- The maximum message length *maxMsgLenBytes*.
- The length of the encoding buffer, *bufferLenBits*

**Inputs**:

- The message *m*, which is an octet string of length *l* octets
- The public key *h*
- A string *s* as a the source of randomness.

**Output**: The ciphertext *c*, which is a ring element, or "message too long"

**Operation**: The ciphertext *e* shall be calculated by the following or an equivalent sequence of steps:

a) If *lLen* > *maxLen*, output "message too long" and stop.
b) Form the octet string p0, consisting of the 0 byte repeated (*maxMsgLenBytes* + 1 - *lLen*) times.
c) Form the octet string *M* of length *bufferLenBits*/8 as *m* ||*p0*||*b*|| *octL* .
d) Convert *M* to a bit string *Mbin* using OS2BSP. Parse it as a degree *8lLen* polynomial.
e) Pop a *sLen* length of string from *s* as the salt *b*. Parse *b* as a degree *8sLen* polynomial.
f) Compute $m = Mbin + b * X^{N-8sLen-1}$
g) Convert the public key *h* to a bit string *bh* using RE2BSP (7.5.1). Form the bit string *bhTrunc* by taking the first *pkLen* bits of *bh*. Convert *bhTrunc* to the octet string *hTrunc*, of length *pkLen*/8 using BS2OSP. Form *sData* as the octet string *m* || *b* || *hTrunc*
h) Use the discrete Gaussian polynomial generator with the seed *sData* and parameters N, dr to produce two Gaussian polynomials *r* and *e*.
i) Calculate $R = r \times h \bmod q$, and $Rp = R \bmod p$
j) Use the random binary polynomial generation function with the seed *Rp* and the chosen parameters to produce a polynomial *mask*.
k) Form m' by polynomial addition of *m* and *mask* mod p.
l) If the number of 1s or 0s in m' is less than $d_{m0}$, discard m' and return to step b).
m) Calculate the ciphertext as $c = R + p*e + m' \bmod q$.
n) Output *c*.

### 12.1.3 Decryption Operation

This clause defines the decryption operation.

**Components**:

- The length of the input ciphertext *c*, *cLen* in bytes.
- The length of the salt *b*, *sLen* in bytes.
- The fixed weight polynomial generation function
- The number of bits of public key to hash, *pkLen*.
- The lower bound *A*
- The minimum message representative weight $d_{m0}$
- The maximum message length *maxMsgLenBytes*

**Inputs**:

- The ciphertext *c*, which is a polynomial of degree *N*-1.
- The private key *F* .
- The public key *h*

**Output**: The message *m*, which is an octet string, or "fail".

**Operation**: The message m shall be calculated by the following or an equivalent sequence of steps:

a) Calculate:
   1) *nLen* = ceil [*N*/8], the number of octets required to hold *N* bits.
   2) *bLen* = *db*/8, the length in octets of the random data
   3) *maxLen* = *nLen* - 1 - *lLen* - *bLen*, the maximum message length.
b) Compute *f* = *pF+1*
c) Decrypt the ciphertext *e* using the selected NTRU decryption primitive with inputs *c* and *f* to get the candidate decrypted binary polynomial *cm'*.
d) If the number of 1s or 0s in *ci* is less than $d_{m0}$, set "fail" to 1.
e) Calculate the candidate value for $r \times h + e$ , *cR* = *c* - *cm' mod q*, and candidate *cRp* = *cR* mod *p*.
o) Use the discrete Gaussian polynomial generation function with the seed *cRp* and the chosen parameters to produce a polynomial *mask*
f) Form *cM* by polynomial subtraction of *cm'* and *mask* mod *p*.
g) Parse *cM* as follows.
   1) The first *lLen* octets represent the message length. Convert the value stored in these octets to the candidate message length *cl*. If *cl* > *maxMsgLenBytes*, set *fail* = 1 and set *cl* = *maxL*.
   2) The next *cl* octets are the candidate message *cm*. the remaining octets should be 0. If they are not, set fail = 1.
   3) The last *bLen* octets are the candidate salt *cb*.
h) Convert the public key *h* to a bit string *bh* using RE2BSP (7.5.1). Form the bit string *bhTrunc* by taking the first *pkLen* bits of *bh*. Convert *bhTrunc* to the octet string *hTrunc*, of length *pkLen*/8 using BS2OSP. Form *sData* as the octet string *cm* || *b* || *hTrunc*
i) Use the discrete Gaussian polynomial generation function with the seed *sData* and parameters N, sigma to produce two polynomials *cr* and *ce*
j) Calculate *cR*' = $h \times cr + ce$ mod *q*.
k) If *cR*' != *cR*, set *fail* = 1
l) If *fail* = 1, output "fail". Otherwise, output *cm* as the decrypted message *m*.

### 12.1.4 Key Pair Validation Methods

Same as NTRU-KEM key pair validation methods in section 11.1.4.

### 12.1.5 Public-key validation

Same as NTRU-KEM public key pair validation methods in section 11.1.5.

## 12.2 Possible Parameter Sets

| Parameter set | p | q | N | max msg length | sigma | pk len | sk len |
|---|---|---|---|---|---|---|---|
| SS-NTRU-CCA-1024 | 2 | 1073750017 | 1024 | 95 | 724 | 4097 | 4097 |

# 13 Digital signature scheme

The following clause defines the supported digital signature schemes. The only signature scheme currently supported is pqNTRUSign. It is also known as NTRU Modular Lattice Signature (NTRUMLS) scheme.

## 13.1 Signature Scheme Operations

The pqNTRUSign scheme consists of the five operations: key generation, key pair validation, public key validation, signing and verification. These operations are defined generally in this clause without assuming any specific choices of the Components listed in Clause Signature Scheme Overview.

### 13.1.1 Key Generation

A key pair shall be generated using the following or a mathematically equivalent set of steps. Note that the algorithm below outputs only the values $f$ and $h$. In some applications it may be desirable to store the values $f^{-1}$ and $g$ as well. This standard does not specify the output format for the key as long as it is unambiguous

**Components:**

- The parameters $N$, $q$, $p$, $dF$, $d_g$
- Fixed weight polynomial generation function.

**Input**: A string $s$ as a the source of randomness.

**Output:** A key pair consisting of the private key $(f, g)$ and the public key $h$

**Operation:** The key pair shall be computed by the following or an equivalent sequence of steps:

a) Call the fixed weight polynomial generator of section 6.4.1.2 with input $N$, $dF$, $dF$, $s$ to obtain a trinary polynomial $F$.
b) Compute the polynomial $f := 1 + p \times F$ in the ring.
c) Compute the polynomial $f^{-1}$ (i.e. the polynomial $f^{-1}$ such that $f^{-1} \times f = f \times f^{-1} = 1$) in $(\mathbf{Z}/q\mathbf{Z})[X]/(X^N - 1)$. If $f^{-1}$ does not exist, go to step a).

d) Call the fixed weight polynomial generator of section 6.4.1.2 with input $N$, $dg$, $dg$, $s$ to obtain a trinary polynomial $g$.

e) Set $g = g+1$.

f) Compute the polynomial $g^{-1}$ (i.e. the polynomial $g^{-1}$ such that $g^{-1} \times g = g \times g^{-1} = 1$) in $(\mathbf{Z}/q\mathbf{Z})[X]/(X^N - 1)$. If $g^{-1}$ does not exist, go to step d).

g) Compute the polynomial $h := f^{-1} \times g \times p$ in the ring.

h) Output $f$, $g$, $h$.

### 13.1.2 Signing Operation

This clause defines the signing operation.

**Components**:

- The OID, an octet string
- Fixed weight polynomial generation function.
- Uniform random polynomial generation function.
- An approved hash function Hash()

**Inputs**:

- The message $m$, which is an octet string of length $l$ octets
- Parameters $N$, $p$, $B_s$, $B_t$, sigma, M, and method.
- The secret key $f$, $g$, $g^{-1}$
- The public key $h$ and its length $pk\_len$
- A string $s$ as a the source of randomness.

**Output**: A transcript of signature, which is an octet string

**Operation**: A transcript of signature $s$ shall be calculated by the following or an equivalent sequence of steps:

a) Call the hash function with input $(m|h)$ and length $l+pk\_len$ to obtain a message digest $msg\_digest$;

b) Call the uniform random polynomial generation function with msg_digest and modulus $p = 2$ to obtain two uniform mod $p$ polynomials $s_p$ and $t_p$

c) if method = uniform
   1) Use the uniform random polynomial generator with input N, $-q/2p+1/2$, seed $s$ and the chosen parameters, to generate a polynomial $r$.
   2) set $b = 0$.

d) if method = Gaussian
   1) Use the discrete Gaussian polynomial generator with input N, sigma, seed $s$ and the chosen parameters, to generate a polynomial $r$;
   2) Generate a random bit $b$.

e) Compute
   1) $s_0 = p \times r + s_p$;
   2) $t_0 = s_0 \times h$;
   3) $a = g^{-1} \times (t_p - t_0) \bmod p$;
   4) $s = s_0 + (-1)^b\ a \times f$;
   5) $t = t_0 + (-1)^b\ a \times g$.

f) Set $s_1 = (s - s_p)/p$.

g) if method = uniform

    1) Calculate the infinity-norms $\|s\|_{\square}$, $\|t\|_{\square}$ as specified in 6.3. If $\|s\|_{\square} > q/2 - B_s$ or $\|t\|_{\square} > q/2 - B_t$, go to c).

h) if method = Gaussian

    1) With probability $1/(M \cdot \exp - \frac{\|af\|}{2\sigma^2} \cosh \frac{\langle s_1, af \rangle}{\sigma^2})$, proceed; otherwise, go to d)
    2) Calculate the infinity-norms $\|t\|_{\square}$ as specified in 6.3. If $\|t\|_{\square} > q/2 - B_t$, go to d).

i) Use the RE2OSP to convert $s_1$ into an octet string *str*.

j) Output *str*.

### 13.1.3 Verification Operation

This clause defines the verification operation.

**Components**:

- The OID, an octet string
- The chosen Coefficient Generation Function and the associated parameters
- An approved hash function Hash().

**Inputs**:

- The message *m*, which is an octet string of length *l* octets.
- A signature *str,* which is an octet string.
- The public key *h*.
- Parameters $N$, $p$, $B_s$ and $B_t$.

**Output**: “Accept” if the signature is valid, or "Reject" if not.

**Operation**: The verification shall be proceeded by the following or an equivalent sequence of steps:

a) Use the OS2REP to convert *str* into a ring element $s_1$.

b) Call the hash function with input $(m|h)$ and length $l+pk\_len$ to obtain a message digest *msg_digest;*

c) Call the uniform random polynomial generation function with msg_digest and modulus $p = 2$ to obtain two uniform mod $p$ polynomials $s_p$ and $t_p$

d) Compute $s = ps_1 + s_p$,

    1) if method = uniform, check if $\|s\|_{\square} > q/2 - B_s$, output “Reject” if yes.
    2) if method = Gaussian, check if $\|s\|_{\square} > 14\ sigma$, output “Reject” if yes.

e) Compute $t = s \times h$, check if $\|t\|_{\square} > q/2 - B_t$, output “Reject” if yes.

f) Check if $t_p = t \bmod p$, output “Reject” if not.

g) Output “Accept”.

### 13.1.4 Key Pair Validation Methods

A same key pair validation method as described in 11.1.4.

### 13.1.5 Public-key validation

A same key pair validation method as described in 11.1.5.

## 13.2 Possible Parameter Sets

### 13.2.1 General

This section defines specific sets of parameters for pqNTRUSign that give a specific level of security according to the metrics in this standard.

| Parameter set | p | q | N | Std. dev | df | dg | pk len | sig len | Bs bound | Bt bound |
|---|---|---|---|---|---|---|---|---|---|---|
| pqNTRUSign-Uniform-512 | 2 | 65537 | 512 | n/a | 77 | 77 | 1041 | 1041 | 40 | 40 |
| pqNTRUSign-Gaussian-512 | 2 | 65537 | 512 | 107 | 77 | 77 | 1041 | 1041 | 215 | 40 |

# 14 ASN.1 Syntax

This section covers the representation of cryptographic objects used in NTRUEncrypt in terms of ASN.1 Syntax. This is important for use with certificates, certificate revocation and other cryptographic messages. In particular, ASN.1 syntax is used to represent the contents of X.509 certificates. Some additional object identifiers and placeholders for ASN.1 syntax for NTRUSign are included in the ASN.1 module in Annex A for informational purposes. ASN.1 and associated encoding rules are defined in [B17][B18][B19][B20][B21][B22][B23][B24][B25].

## 14.1 General Types

### 14.1.1 General Vector Types

This section defines the ASN.1 syntax for vector types that are used to represent polynomials for NTRUEncrypt. There are four primary types of vectors – public vectors, binary vectors, trinary vectors, and listed vectors. Public vectors are polynomials that have $N$ coefficients that are reduced modulo $q$. Binary vectors are polynomials that have $N$ coefficients that are reduced modulo $p$, where $p = 2 + X$, making the coefficients all either 0 or 1. Trinary vectors are polynomials that have $N$ coefficients that are reduced modulo $p$, where $p = 3$, making the coefficients either 0, 1 or –1. Coefficients in these vectors are always represented as positive integers, however since the coefficients are taken modulo either $p$ or $q$, they should be reduced into the appropriate interval before being used (e.g. modulo 3 numbers are reduced to 0, 1 or -1 and modulo $q$ numbers are

usually reduced into the interval $-q/2 <= x <= q/2$). Finally, listed vectors are an array of indices mod $N$, where each index identifies a coefficient with a known value.

NOTE: Binary vectors are not supported in this version of the standard but are included for backwards compatibility of the ASN.1 structures.

All of the vector types consist of a string of integer values that are concatenated and stored in an OCTET STRING. Each integer is encoded by taking the smallest positive representation of the integer modulo $p$, $q$ or $N$ (e.g. taking –1 as a mod 3 number gives the integer 2) and using I2BSP (see section7.2.1) to obtain a bit string of the appropriate length. The integer is recovered by obtaining the correct bit string (e.g. for the NTRUEncrypt parameters in this standard, each coefficient of a polynomial mod q = 2048 is represented by 11 bits), and using BS2IP (see section 7.2.2). So, to encode the value 55 as an 11-bit value, the integer is encoded as the 11-bit string 000 0011 0111 (using I2BSP). To obtain the value of the coefficient represented by the bit string 000 0011 0111, the bit string is converted to the integer 55 (using BS2IP).

```
NTRUPublicVector ::= CHOICE {
        modQVector              ModQVector, -- not recommended
        packedModQVector        PackedModQVector,
        …
}

NTRUBinaryVector ::= CHOICE {
        listedBinaryVector      ListedBinaryVector,
        packedBinaryVector      PackedBinaryVector,
        modQVector              ModQVector,
        …
}
```

Binary vectors are not supported in this version of the standard.

**ModQVector ::= OCTET STRING**

The contents of this **OCTET STRING** are obtained by converting a ring element to an octet string using the RE2POSP conversion primitive. A **ModQVector** is a representation of a polynomial of degree $N$ and must include all $N$ coefficients, even if the high-order ones are zero.

**PackedModQVector ::= OCTET STRING**

The contents of this **OCTET STRING** are obtained by converting a ring element to an octet string using the RE2OSP conversion primitive. A **PackedModQVector** is a representation of a polynomial of degree $N$ and must include all $N$ coefficients, even if the high-order ones are zero.

This is the preferred format for public keys and ciphertexts for the parameter sets in this standard.

**ListedBinaryVector ::= OCTET STRING**

This type is not supported in this version of this standard.

**PackedBinaryVector ::= OCTET STRING**

This type is not supported in this version of this standard.

**ListedTrinaryVector ::= OCTET STRING**

The contents of this **OCTET STRING** are obtained by converting a trinary vector to an octet string with TRE2OSP. This is the preferred format for NTRUEncrypt private key components.

**PackedTrinaryVector ::= OCTET STRING**

The contents of this **OCTET STRING** are obtained by converting a trinary vector to an octet string with RE2OSP with q set equal to 3.

We define two further types, which can be used to represent a polynomial of arbitrary degree:

Object Identifiers
This standard uses the following base object identifiers.

```
ntru OBJECT IDENTIFIER ::= {
        iso(1) identified-organization(3) dod(6) internet(1) private(4) enterprises(1)
        ntruCryptosystems (8342) }

        id-eess1 OBJECT IDENTIFIER ::= { ntru eess (1) eess-1 (1) }

id-eess1-algs OBJECT IDENTIFIER ::= {id-eess1  1}
id-eess1-params OBJECT IDENTIFIER ::= {id-eess1  2}
id-eess1-encodingMethods OBJECT IDENTIFIER ::= {id-eess1 3}
```

## *14.2 ASN.1 for NTRUEncrypt SVES*

This section defines the ASN.1 object identifiers for NTRUEncrypt keys and NTRUEncrypt encrypted data, and defines the types **NTRUPublicKey**, **NTRUPrivateKey**, **NTRUEncryptedData**, and **EESS1v1-SVES-Parameters**.

The object identifier **id-ntru-EESS1v1-SVES** identifies NTRUEncrypt public and private keys and NTRUEncrypt-encrypted data. When this object identifier is used in an **AlgorithmIdentifier**, the parameters shall be of type **EESS1v1-SVES-Parameters**.

Note that EESS#1 breaks with common practice in requiring that a key be encoded with the scheme parameters (such as a mask generation function identifier for NTRUEncrypt

or the verification bounds for NTRUSign) as well as with the algorithm domain parameters (such as *N*, *q* and *p*). Ensuring that a key can only be used in one scheme provides a defense against version rollback attacks and is good security practice.

This section of this standard only defines ASN.1 for the currently supported parameter sets. ASN.1 for previously parameter sets will appear in a future appendix to this standard.

### 14.2.1 NTRUEncrypt Public Keys

NTRUEncrypt public keys are identified by the following object identifier:

**id-ntru-EESS1v1-SVES OBJECT IDENTIFIER ::= {id-eess1-algs 1}**

The parameters field associated with this OID in an **AlgorithmIdentifier** shall have the type **EESS1v1-SVES-params**, defined in section 11.2.4 below.

NTRUEncrypt public keys should be represented with the following syntax:

```
NTRUPublicKey ::= SEQUENCE {
        publicKeyVector         NTRUPublicVector,  -- h
        ntruKeyExtensions       NTRUKeyExtensions OPTIONAL
        }

NTRUKeyExtensions ::= SEQUENCE SIZE(1..MAX) OF NTRUKeyExtension

NTRUKeyExtension ::= CHOICE {
        keyID           INTEGER,
        …}
```

The fields of the type **NTRUPublicKey** have the following meanings:

- **NTRUPublicKeyVector** is the polynomial h.
- **NTRUKeyExtensions** is provided for future extensibility. Only one extension is defined in EESS#1.

The fields of the type **NTRUKeyExtension** have the following meanings:

- **keyID** can be used to associate a unique key identifier with the key.

### 14.2.2 NTRUEncrypt Private Keys

NTRUEncrypt private keys are identified by the following object identifier:

**id-ntru-EESS1v1-SVES OBJECT IDENTIFIER ::= {id-eess1-algs 1}**

They are distinguished from NTRUEncrypt public keys by form and by context. The parameters field associated with this OID in an **AlgorithmIdentifier** shall have the type **EESS1v1-SVES-params**, defined in section 11.2.4 below.

An NTRUEncrypt private key should be represented with the following syntax:

```
NTRUTrinaryPrivateKey ::= SEQUENCE {
Version                  INTEGER,
publicKeyVector          NTRUPublicVector OPTIONAL,
ntruPrivateKeyVectors CHOICE {
          productForm    NTRUProductFormTrinaryPrivateKeyVectors,
          …
       },
        …}
```

```
NTRUProductFormTrinaryPrivateKeyVectors ::= SEQUENCE {
f1                       ListedTrinaryVector,
f2                       ListedTrinaryVector,
f3                       ListedTrinaryVector,
g                        PackedTrinaryVector OPTIONAL }
```

The fields of the type **NTRUPrivateKey** have the following meanings:

- **version** is the version number, for compatibility with future revisions of this document. It shall be 0 for this version of the document.
- **publicKeyVector** is the public key associated with the private key. To complete the ciphertext validity check when decrypting, the decrypter must know the public key. It can be provided either explicitly in this field, or implicitly by providing the **GVectors** in the **ntruPrivateKeyVectors** field.
- **privateKeyVectors** contains the private key vector. The only type of private key vector supported in this standard has $f = 1 + p*(f1*f2 + f3)$.

### 14.2.3 NTRUEncrypt Encrypted Data

NTRUEncrypt encrypted data are identified by the following object identifier:

**id-ntru-EESS1v1-SVES OBJECT IDENTIFIER ::= {id-eess1-algs 1}**

The parameters field associated with this OID in an **AlgorithmIdentifier** shall have the type **EESS1v1-SVES-params**, defined in section 11.2.4 below.

NTRUEncrypt encrypted data should be represented with the **NTRUEncryptedData** type:

**NTRUEncryptedData ::= NTRUPublicVector**

The preferred format for **NTRUEncryptedData** is a **PackedModQVector**.

### 14.2.4 NTRUEncrypt Parameters

This section defines the parameters associated with the **id-ntru-EESS1v1-SVES** OID in an **AlgorithmIdentifier**. These parameters shall have type **EESS1v1-SVES-Parameters**:

```
EESS1v1-SVES-Parameters ::= CHOICE {
        degree                      INTEGER, -- this choice is deprecated
        standardNTRUParameters      StandardNTRUParameters,
        explicitNTRUParameters      ExplicitNTRUParameters,
        externalParameters          NULL }

StandardNTRUParameters ::= OIDS.&id({NTRUParameters})

NTRUParameters OIDS ::= {
        { OID id-ees401ep2 } |
        { OID id-ees439ep1 } |
        { OID id-ees593ep1 } |
        { OID id-ees743ep1 } |
        { OID id-ees443ep1 } |
        { OID id-ees587ep1 } |
        … -- allows for future expansion
        -- other OIDs defined in previous versions of this standard are deprecated
        }

id-ees401ep2 OBJECT IDENTIFIER ::= {id-eess1-params 46}
id-ees439ep1 OBJECT IDENTIFIER ::= {id-eess1-params 47}
id-ees593ep1 OBJECT IDENTIFIER ::= {id-eess1-params 48}
id-ees743ep1 OBJECT IDENTIFIER ::= {id-eess1-params 49}
id-ees443ep1 OBJECT IDENTIFIER ::= {id-eess1-params 50}
id-ees587ep1 OBJECT IDENTIFIER ::= {id-eess1-params 51}
```

- **degree** is deprecated
- **standardNTRUParameters** identifies the parameters by use of an OID. In this document, six OIDs are defined: **id-ees401ep2**, **id-ees439ep1**, **id-ees593ep1**, **id-ees743ep1**, **id-ees443ep1**, and **id-ees587ep1**.
- **explicitNTRUParameters** allows an implementer to specify parameter sets other than those specified in this document. It is not supported in this version of this document.
- **externalParameters** should be used if the parameters are being inherited from some other source (for example, in X.509 certificates, if the parameters are being inherited from the CA's parameters).

## *14.3 ASN.1 for pqNTRUSign*

This section defines the ASN.1 object identifiers for pqNTRUSign keys and pqNTRUSign digital signature data, and defines the types **pqNTRUSignPublicKey**, **pqNTRUSignPrivateKey**, **pqNTRUSignSignature**, and **EESS1v1-pqNTRUSign-Parameters**.

The object identifier **id-ntru-EESS1v1-pqNTRUSign** identifies pqNTRUSign public and private keys and pqNTRUSign digital signature data. When this object identifier is used

in an **AlgorithmIdentifier**, the parameters shall be of type **EESS1v1-pqNTRUSign-Parameters**.

Note that EESS#1 breaks with common practice in requiring that a key be encoded with the scheme parameters (such as a mask generation function identifier for NTRUEncrypt or the verification bounds for pqNTRUSign) as well as with the algorithm domain parameters (such as *N*, *q* and *p*). Ensuring that a key can only be used in one scheme provides a defense against version rollback attacks and is good security practice.

This section of this standard only defines ASN.1 for the currently supported parameter sets. ASN.1 for previously parameter sets will appear in a future appendix to this standard.

### 14.3.1 pqNTRUSign Public Keys

pqNTRUSign public keys are identified by the following object identifier:

**id-ntru-EESS1v1-pqNTRUSign OBJECT IDENTIFIER ::= {id-eess1-algs 4}**

The parameters field associated with this OID in an **AlgorithmIdentifier** shall have the type **EESS1v1-pqNTRUSign-params**, defined in section 11.2.4 below.

pqNTRUSign public keys should be represented with the following syntax:

```
pqNTRUSignPublicKey ::= SEQUENCE {
        publicKeyVector        pqNTRUSignPublicVector,  -- h
        ntruKeyExtensions      pqNTRUSignKeyExtensions OPTIONAL
        }

pqNTRUSignKeyExtensions ::= SEQUENCE SIZE(1..MAX) OF pqNTRUSignKeyExtension

pqNTRUSignKeyExtension ::= CHOICE {
        keyID            INTEGER,
        ...}
```

The fields of the type **pqNTRUSignPublicKey** have the following meanings:

- **pqNTRUSignPublicKeyVector** is the polynomial h.
- **pqNTRUSignKeyExtensions** is provided for future extensibility. Only one extension is defined in EESS#1.

The fields of the type **pqNTRUSignKeyExtension** have the following meanings:

- **keyID** can be used to associate a unique key identifier with the key.

### 14.3.2 pqNTRUSign Private Keys

pqNTRUSign private keys are identified by the following object identifier:

**id-ntru-EESS1v1- pqNTRUSign OBJECT IDENTIFIER ::= {id-eess1-algs 4}**

They are distinguished from pqNTRUSign public keys by form and by context. The parameters field associated with this OID in an **AlgorithmIdentifier** shall have the type **EESS1v1- pqNTRUSign-params**, defined in section 11.2.4 below.

A pqNTRUSign private key should be represented with the following syntax:

```
pqNTRUSign TrinaryPrivateKey ::= SEQUENCE {
version                              INTEGER,
publicKeyVector         pqNTRUSignPublicVector OPTIONAL,
ntruPrivateKeyVectors CHOICE {
          productForm      pqNTRUSignProductFormTrinaryPrivateKeyVectors,
          …
     },
     …}
```

```
pqNTRUSignProductFormTrinaryPrivateKeyVectors ::= SEQUENCE {
f1                                   ListedTrinaryVector,
f2                                   ListedTrinaryVector,
f3                                   ListedTrinaryVector,
g                         PackedTrinaryVector OPTIONAL }
```

(In the printed `g` line, "OPTIONAL" is struck through.)

The fields of the type **pqNTRUSignTrinaryPrivateKey** have the following meanings:

- **version** is the version number, for compatibility with future revisions of this document. It shall be 0 for this version of the document.
- **publicKeyVector** is the public key associated with the private key. To complete the ciphertext validity check when decrypting, the decrypter must know the public key. It can be provided either explicitly in this field, or implicitly by providing the **GVectors** in the **ntruPrivateKeyVectors** field.
- **ntruPrivateKeyVectors** contains the private key vector. The only type of private key vector supported in this standard has f = 1 + $p$*(f1*f2 + f3).

### 14.3.3 pqNTRUSign Signature Data

pqNTRUSign signature data are identified by the following object identifier:

**id-ntru-EESS1v1- pqNTRUSign OBJECT IDENTIFIER ::= {id-eess1-algs 4}**

The parameters field associated with this OID in an **AlgorithmIdentifier** shall have the type **EESS1v1- pqNTRUSign-params**, defined in section 11.2.4 below.

pqNTRUSign signature data should be represented with the **pqNTRUSignSignature** type:

**pqNTRUSignSignature ::= NTRUPublicVector**

The preferred format for **pqNTRUSignSignature** is a **PackedModQVector**.

### 14.3.4 pqNTRUSign Parameters

This section defines the parameters associated with the **id-ntru-EESS1v1-pqNTRUSign** OID in an **AlgorithmIdentifier**. These parameters shall have type **EESS1v1-pqNTRUSign-Parameters**:

```
EESS1v1-pqNTRUSign-Parameters ::= CHOICE {
        degree                      INTEGER, -- this choice is deprecated
        standardNTRUParameters      StandardPqNTRUSignParameters,
        explicitNTRUParameters      ExplicitPqNTRUSignParameters,
        externalParameters          NULL }

StandardpqNTRUSignParameters ::= OIDS.&id({pqNTRUSignParameters})

pqNTRUSignParameters OIDS ::= {
        { OID id-mls-443ep1 } |
        { OID id-mls-563ep1 } |
        { OID id-mls-743ep2 } |
        { OID id-mls-907ep1 } |
        … -- allows for future expansion
        -- other OIDs defined in previous versions of this standard are deprecated
        }

id-mls-443ep1 OBJECT IDENTIFIER ::= {id-eess1-params 6}
id-mls-563ep1 OBJECT IDENTIFIER ::= {id-eess1-params 7}
id-mls-743ep2 OBJECT IDENTIFIER ::= {id-eess1-params 8}
id-mls-907ep1 OBJECT IDENTIFIER ::= {id-eess1-params 9}
```

- **degree** is deprecated
- **standardPqNTRUSignParameters** identifies the parameters by use of an OID. In this document, four OIDs are defined: **id-mls-443ep1, id-mls-563ep1, id-mls-743ep2 and id-mls-907ep1**.
- **explicitPqNTRUSignParameters** allows an implementer to specify parameter sets other than those specified in this document. It is not supported in this version of this document.
- **externalParameters** should be used if the parameters are being inherited from some other source (for example, in X.509 certificates, if the parameters are being inherited from the CA's parameters).

## Appendix A - NTRU ASN.1 Module

```
EESS-1 {iso(1) identified-organization(3) dod(6) internet(1) private(4) enterprises(1)
ntruCryptosystems(8342) eess(1) eess-1(1) modules(0) eess-1(1)}

-- $ revision: 3.0 $

DEFINITIONS IMPLICIT TAGS ::= BEGIN

-- EXPORTS All; --
-- All types and values defined in this module are exported for use in other ASN.1 modules.

-- IMPORTS None; --

-- Supporting definitions

AlgorithmIdentifier { ALGORITHM: IOSet } ::= SEQUENCE {
        algorithm      ALGORITHM.&id({IOSet}),
        parameters     ALGORITHM.&Type({IOSet}{@algorithm}) OPTIONAL
}

ALGORITHM ::= CLASS {
        &id      OBJECT IDENTIFIER  UNIQUE,
        &Type  OPTIONAL
}
        WITH SYNTAX { OID &id [PARMS &Type] }

OIDS ::= ALGORITHM

-- Informational object identifiers

pkcs-1 OBJECT IDENTIFIER ::= {
        iso(1) member-body(2) us(840) rsadsi(113549) pkcs(1) 1 }

id-mgf1 OBJECT IDENTIFIER ::= { pkcs-1 8}

id-sha1 OBJECT IDENTIFIER ::= {
        iso(1) identified-organization(3) oiw(14) secsig(3) algorithms(2) 26 }

id-sha256 OBJECT IDENTIFIER ::= {
joint-iso-itu-t(2) country(16) us(840) organization(1) gov(101)
csor(3)  nistalgorithm(4) hashalgs(2) 1 }

id-sha384 OBJECT IDENTIFIER ::= {
joint-iso-itu-t(2) country(16) us(840) organization(1) gov(101)
csor(3)  nistalgorithm(4) hashalgs(2) 2 }

id-sha512 OBJECT IDENTIFIER ::= {
joint-iso-itu-t(2) country(16) us(840) organization(1) gov(101)
csor(3)  nistalgorithm(4) hashalgs(2) 3 }
```

```
-- Basic object identifiers

ntru OBJECT IDENTIFIER ::= {
iso(1) identified-organization(3) dod(6) internet(1) private(4) enterprises(1)
ntruCryptosystems (8342) }

id-eess1 OBJECT IDENTIFIER ::= { ntru eess (1) eess-1 (1) }

id-eess1-algs OBJECT IDENTIFIER ::= {id-eess1  1}
id-eess1-params OBJECT IDENTIFIER ::= {id-eess1  2}
id-eess1-encodingMethods OBJECT IDENTIFIER ::= {id-eess1 3}

-- algorithms

id-ntru-EESS1v1-SVES OBJECT IDENTIFIER  ::= {id-eess1-algs  1}
id-ntru-EESS1v1-pqNTRUSign OBJECT IDENTIFIER  ::= {id-eess1-algs  4}


-- parameter set identifiers

id-ees401ep2 OBJECT IDENTIFIER ::= {id-eess1-params 46}
id-ees439ep1 OBJECT IDENTIFIER ::= {id-eess1-params 47}
id-ees593ep1 OBJECT IDENTIFIER ::= {id-eess1-params 48}
id-ees743ep1 OBJECT IDENTIFIER ::= {id-eess1-params 49}
id-ees443ep1 OBJECT IDENTIFIER ::= {id-eess1-params 50}
id-ees587ep1 OBJECT IDENTIFIER ::= {id-eess1-params 51}

id-mls-443ep1 OBJECT IDENTIFIER ::= {id-eess1-params 6}
id-mls-587ep1 OBJECT IDENTIFIER ::= {id-eess1-params 7}
id-mls-743ep2 OBJECT IDENTIFIER ::= {id-eess1-params 8}
id-mls-907ep1 OBJECT IDENTIFIER ::= {id-eess1-params 9}

-- General types

ModQVector ::= OCTET STRING

PackedModQVector ::= OCTET STRING

ListedBinaryVector ::= OCTET STRING

PackedBinaryVector ::= OCTET STRING

ListedTrinaryVector ::= OCTET STRING

PackedTrinaryVector ::= OCTET STRING

-- NTRUEncrypt Encryption

NTRUPublicVector ::= CHOICE {
        modQVector          ModQVector, -- not recommended
```

```
        packedModQVector     PackedModQVector,
    ...
}

NTRUKeyExtension ::= CHOICE {
        keyID              INTEGER,
        ...}

NTRUPublicKey ::= SEQUENCE {
        publicKeyVector  NTRUPublicVector,
        ntruKeyExtensions      SEQUENCE SIZE (1..MAX) OF
NTRUKeyExtension OPTIONAL }

NTRUTrinaryPrivateKey ::= SEQUENCE {
        version                INTEGER,
        publicKeyVector        NTRUPublicVector OPTIONAL,
        ntruPrivateKeyVectors CHOICE {
          productForm              NTRUProductFormTrinaryPrivateKeyVectors,
          ...
        },
...}

NTRUProductFormTrinaryPrivateKeyVectors ::= SEQUENCE {
        f1                 ListedTrinaryVector,
        f2                 ListedTrinaryVector,
        f3                 ListedTrinaryVector,
        g                  PackedTrinaryVector OPTIONAL }

NTRUEncryptedData ::= NTRUPublicVector

EESS1v1-SVES-Parameters ::= CHOICE {
        degree                        INTEGER, -- this choice is deprecated
        standardNTRUParameters        StandardNTRUParameters,
        explicitNTRUParameters        ExplicitNTRUParameters,
        externalParameters            NULL }

StandardNTRUParameters ::= OIDS.&id({NTRUParameters})

NTRUParameters OIDS ::= {
{ OID id-ees401ep2 } |
{ OID id-ees439ep1 } |
{ OID id-ees593ep1 } |
{ OID id-ees743ep1 } |
{ OID id-ees443ep1 } |
{ OID id-ees587ep1 } |
        … -- allows for future expansion
        -- other OIDs defined in previous versions of this standard are deprecated
        }

ExplicitNTRUParameters ::= OCTET STRING

-- pqNTRUSign Signature
```

```
pqNTRUSignPublicVector ::= CHOICE {
        modQVector          ModQVector, -- not recommended
        packedModQVector    PackedModQVector,
   ...
}

pqNTRUSignKeyExtension ::= CHOICE {
        keyID           INTEGER,
        ...}

pqNTRUSignPublicKey ::= SEQUENCE {
        publicKeyVector  pqNTRUSignPublicVector,
        ntruKeyExtensions     SEQUENCE SIZE (1..MAX) OF
NTRUKeyExtension OPTIONAL }

pqNTRUSignTrinaryPrivateKey ::= SEQUENCE {
        version                 INTEGER,
        publicKeyVector         pqNTRUSignPublicVector OPTIONAL,
        ntruPrivateKeyVectors CHOICE {
          productForm             pqNTRUSign ProductFormTrinaryPrivateKeyVectors,
          ...
        },
...}

pqNTRUSignProductFormTrinaryPrivateKeyVectors ::= SEQUENCE {
        f1              ListedTrinaryVector,
        f2              ListedTrinaryVector,
        f3              ListedTrinaryVector,
        g               PackedTrinaryVector OPTIONAL }

pqNTRUSignSignature ::= pqNTRUSignPublicVector

EESS1v1- pqNTRUSign-Parameters ::= CHOICE {
        degree                      INTEGER, -- this choice is deprecated
        standardNTRUParameters      StandardpqNTRUSignParameters,
        explicitNTRUParameters      ExplicitpqNTRUSignParameters,
        externalParameters          NULL }

StandardpqNTRUSignParameters ::= OIDS.&id({pqNTRUSignParameters})

pqNTRUSignParameters OIDS ::= {
        { OID id-mls-443ep1 } |
        { OID id-mls-563ep1 } |
        { OID id-mls-743ep2 } |
        { OID id-mls-907ep1 } |
        … -- allows for future expansion
        -- other OIDs defined in previous versions of this standard are deprecated
        }

ExplicitpqNTRUSignParameters ::= OCTET STRING

END  -- EESS-1
```

# Appendix B - Security Considerations

## *B.1 Security against classical computers*

Security analysis for the recommended parameter sets is provided in [B11].

In addition to this analysis, note that the factorization of the ideal generated by 2 in the $N$th cyclotomic field affects the probability that a public key in dimension $N$ will be invertible. There are no known attacks based on this factorization and a paranoid user can easily check the invertibility of $g$ at key generation time (or of $h$ when encrypting) but to be conservative this version of this standard introduces and prefers parameter sets at $N$=443 rather than $N$=439 and N=587 rather than N=593.

## *B.2 Security against quantum computers*

The use of Grover's algorithm as a subroutine in classical lattice sieving algorithms has been shown, under reasonable assumptions, to provide an asymptotic speedup in solving the shortest vector problem [B27]. However, for the lattice dimensions relevant to standard NTRU parameter sets, these algorithms are significantly outperformed by classical techniques, such as enumeration, for which no quantum speedup has been shown. Attacks which exploit the exceptional algebraic structure of NTRU lattices to obtain significant speedups have also not been forthcoming.

The standard NTRU parameter sets were designed to resist the hybrid attack proposed by Howgrave-Graham [B15]. The hybrid attack balances the cost of lattice reduction based preprocessing against the cost of an exhaustive search in a projected lattice. On a classical computer there is a meet-in-the-middle time/space tradeoff that allows one to square-root the temporal cost of the search phase. Using Grover's algorithm the same temporal speedup can be obtained without the spatial overhead, but one might wonder whether even better by leveraging a time/space tradeoff. Several authors have attempted such tradeoffs [B9][B28], but the claimed speedups in these papers disappear if one properly accounts for the cost of the database queries made by the Grover iteration. At present it appears that the best attack on NTRU is a quantum variant of the hybrid attack that directly replaces the meet-in-the-middle phase with a Grover search. This attack substantially reduces the space required by the hybrid attack, but does not affect the standardized parameter sets as the spatial cost of the hybrid attack was ignored for their analysis.

The full specification of NTRU relies on various symmetric primitives, such as hash functions, and the key-sizes of these must be appropriately tuned to accommodate for quantum-search based speedups. Fluhrer has noted [B9] that the parameter sets ees401ep2 and ees439ep1 make use of SHA-1, and that this entails a reduction in their post-quantum security that could be avoided by simply switching to SHA-256.

Additionally, the use of the salt $b$ to provide semantic security against chosen plaintext attacks may be subject to an attack via Grover’s algorithm: in the case of a classical

computer, the required length of $b$ in bits is equal to the desired bit security level $k$, while under Grover's algorithm a salt of length $2k$ is necessary.

There are attacks that allow one to find short vectors in lattices possessing the structure of a principal ideal of the ring of integers of a cyclotomic field [B4]. These attacks take advantage of the fact that the ideal in question is generated by a single short element and that it is trivial, in cyclotomic fields, to find a short generator given an arbitrary generator. Cryptosystems that rely on the hardness of finding a short generator of a principal ideal only reveal a Z-basis for the ideal, and not a generator, as the public key. The complexity of finding a generator given a Z-basis is super-polynomial with a classical computer but has been shown to be polynomial with a quantum computer; hence these cryptosystems may not be secure against quantum adversaries.

NTRU lattices are modules over a ring of integers, but not ideals, so these attacks do not apply directly. However, there has been a proposal [B3] to embed the module structure of an NTRU lattice into an ideal of the ring of integers of an extension of a cyclotomic field. If the resulting ideal is principal, or if it divides a principal ideal with small quotient, then this embedding may allow an attack like that of [B4]. At present there are a number of open problems related to this attack's complexity and the current parameter recommendations assume that this attack will not have a significant impact on security. Chief among the open problems is the fact that finding the short generator given an arbitrary generator may not be trivial in the extension field, and may involve solving a difficult closest vector problem. An approach to finding a short generator using subfields has been proposed [B2] but appears to require exceptional field structure.

As a very conservative hedge against progress in this direction, NTRU can be instantiated in a ring such as Z[x]/(x^N - x - 1) which carries none of the worrisome structure of the cyclotomic ring. We do not make this recommendation due to the effects on efficiency.

This leads to the recommendation of the parameter sets ees443ep1 for 128-bit pre-quantum security, ees587ep1 for 192-bit pre-quantum security, and ees743ep1 for 256-bit pre-quantum security. All of these parameter sets are also appropriate for the 128-bit security level in the post-quantum computing setting. A conservative user may wish to use ees743ep1 for 128-bit post-quantum security; this can be done with a high degree of confidence.

### *B.3 Rejection sampling for lattice based signatures*

Lattice based signature schemes have a history of almost 20 years. Early lattice based signature schemes, such as GGHSign and NTRUSign, leak private key information in a transcript of message/signature pairs. An attacker can produce a signing key from a long enough transcript using methods for "learning a parallelepiped."

In [B29], Lyubashevsky proposed a rejection sampling method to thwart transcript leakage attacks. Using his technique, signatures are produced according to a fixed public

distribution (typically either a Gaussian or a uniform distribution). A transcript reveals only this public distribution, and contains no information about the particular signing key that is used to generate the signatures. This technique has become the de facto method for avoiding transcript leakage in lattice based signature schemes, such as [B10][B5][B31][B31]. However, to ensure that the output signature follows a given distribution, a large number of random candidate signatures may need to be generated for a single document before one is accepted.

The pqNTRUSign digital signature algorithm described in this document incorporates the rejection sampling technique (see step i) to step k) in section 10.1.2). This ensures the output signature is uniformly distributed over a known distribution, and hence leaks no information about the secret key.

## Appendix B - Test Vectors

[To be added in future versions]

## Appendix C - Revision History

Draft 1.0 available March 27, 2001
Draft 2.0 available May 18, 2001
Draft 3.0 available July 9, 2001
Draft 3.2 available August 30, 2001
Draft 4.0 available March 9, 2002
Draft 5.0 available September 6, 2002
Version 1.0 available November 12, 2002
Version 2.0 available April 14, 2003
Version 3.0 available March 31st, 2015 – includes parameters recommended by [B11]
Version 3.1 available September 15th, 2015 – includes post-quantum security analysis.
Version 3.2 available July 30th, 2017 – includes pqNTRUSign digital signature algorithm.

## Appendix D – References

[B1] ANS X9.71-2000, Keyed Hash Message Authentication Code (MAC).

[B2] D. Bernstein, A subfield-logarithm attack against ideal lattices, available from http://blog.cr.yp.to/20140213-ideal.html.

[B3] Bernstein, D., Soliloquy. Cryptanalytic Algorithms Mailing List. Available at https://groups.google.com/forum/#!topic/cryptanalytic-algorithms/GdVfp5Kbdb8. February 21, 2015.

[B4] Campbell, P., Groves, M., and Shepherd, D. Soliloquy: A cautionary tale. ETSI 2nd Quantum-Safe Crypto Workshop, 2014. Available at http://docbox.etsi.org/Workshop/2014/201410_CRYPTO/S07_Systems_and_Attacks/S07_Groves_Annex.pdf.

[B5] Consortium for Efficient Embedded Security, Efficient Embedded Security Standard (EESS) #1 (https://github.com/NTRUOpenSourceProject/ntru-crypto).

[B6] FIPS PUB 197, Advanced Encryption Standard, Federal Information Processing Standards Publication 186-2, U.S. Department of Commerce/National Institute of Standards and Technology, National Technical Information Service, Springfield, Virginia, Nov 2001. Available at http://csrc.nist.gov/publications/fips/fips197/fips-197.pdf.

[B7] FIPS PUB 180-4, Secure Hash Standard, Federal Information Processing Standards Publication 180-4, U.S. Department of Commerce/National Institute of Standards and Technology, National Technical Information Service, Springfield, Virginia, March, 2012. Available at http://csrc.nist.gov/publications/fips/fips180-4/fips-180-4.pdf.

[B8] FIPS PUB 202 (draft), DRAFT SHA-3 Standard: Permutation-Based Hash and Extendable-Output Functions, Federal Information Processing Standards Publication 202, U.S. Department of Commerce/National Institute of Standards and Technology, National Technical Information Service, Springfield, Virginia, Jul 2013. Available at http://csrc.nist.gov/publications/PubsDrafts.html#FIPS-202.

[B9] Fluhrer, S., "Quantum Cryptanalysis of NTRU." IACR Cryptology ePrint Archive 2015: 676 (2015)

[B10] J. Hoffstein, J. Pipher, J. M. Schanck, J. H. Silverman, W. Whyte, Transcript Secure Signatures Based on Modular Lattices. PQCrypto 2014, available from https://eprint.iacr.org/2014/457.

[B11] J. Hoffstein, J. Pipher, J. M. Schanck, J. H. Silverman, W. Whyte, Z. Zhang, Choosing Parameters for NTRUEncrypt, available from https://eprint.iacr.org/2015/708.

[B12] J. Hoffstein, J. Pipher, J.H. Silverman, NTRU: A Ring Based Public Key Cryptosystem, Algorithmic Number Theory (ANTS III), Portland, OR, June 1998, Lecture Notes in Computer Science 1423, J.P. Buhler (ed.), Springer-Verlag, Berlin, 1998, 267—288

[B13] J. Hoffstein, J. Silverman, Random Small Hamming Weight Products with Applications to Cryptography, Com2MaC Workshop on Cryptography (Pohang, Korea, June 2000), Discrete Mathematics.

[B14] J. Hoffstein, J.H. Silverman, Optimizations for NTRU, Public-Key Cryptography and Computational Number Theory (Warsaw, September 11-15, 2000), DeGruyter.

[B15] N. Howgrave-Graham, A Hybrid lattice reduction and meet-in-the-middle-attack against NTRU. Crypto 2007.

[B16] Nick Howgrave-Graham, Joseph H. Silverman, William Whyte: Choosing Parameter Sets for NTRUEncrypt with NAEP and SVES-3. CT-RSA 2005: 118-135

[B17] ISO/IEC 8824-2:2002, Information technology — Abstract Syntax Notation One (ASN.1): ISO/IEC 8824-2:2002, Information technology — Abstract Syntax Notation One (ASN.1): Specification of basic notation. Also published as ITU-T Recommendation X.680 (2002).

[B18] ISO/IEC 8824-2:2002, Information technology — Abstract Syntax Notation One (ASN.1): Information object specification. Also published as ITU-T Recommendation X.681 (2002).

[B19] ISO/IEC 8824-3:2002, Information technology — Abstract Syntax Notation One (ASN.1): Constraint specification. Also published as ITU-T Recommendation X.682 (2002).

[B20] ISO/IEC 8824-4:2002, Information technology — Abstract Syntax Notation One (ASN.1): Parameterization of ASN.1 specifications. Also published as ITU-T Recommendation X.683 (2002).

[B21] ISO/IEC 8825-1:2002, Information technology — ASN.1 encoding rules: Specification of Basic Encoding Rules (BER), Canonical Encoding Rules (CER) and Distinguished Encoding Rules (DER). Also published as ITU-T Recommendation X.690 (2002).

[B22] ISO/IEC 8825-2:2002, Information technology — ASN.1 encoding rules: Specification of Packed Encoding Rules (PER). Also published as ITU-T Recommendation X.691 (2002).

[B23] ISO/IEC 8825-3:2002, Information technology — ASN.1 encoding rules: Specification of Encoding Control Notation (ECN). Also published as ITU-T Recommendation X.692 (2002).

[B24] ISO/IEC 8825-4:2002, Information technology — ASN.1 encoding rules: XML Encoding Rules (XER). Also published as ITU-T Recommendation X.693 (2002).

[B25] ITU-T Recommendation X.509 (also ISO/IEC 9594-8:1998): Information Technology – Open Systems Interconnection – The Directory: Authentication Framework

[B26] Greg Kuperberg, "A sub-exponential-time quantum algorithm for the dihedral hidden subgroup problem?", 2003, http://arxiv.org/abs/quant-ph/0302112

[B27] Laarhoven, T., Mosca, M., van de Pol, J., "Finding shortest lattice vectors faster using quantum search". Designs, Codes and Cryptography. 2014, p. 1--26.

[B28] Wang, H., Ma, Z., Ma, C., "An efficient quantum meet-in-the-middle attack against NTRU-2005". Chinese Science Bulletin. 2013, vol 58, n. 28-29, p. 3514—3518

[B29] V. Lyubashevsky, Fiat-Shamir with aborts: Applications to lattice and factoring-based signatures. In: ASIACRYPT 2009.

[B30] Ducas, L., Durmus, A., Lepoint, T., Lyubashevsky, V.: Lattice signatures and bimodal Gaussians. In: Canetti, R., Garay, J.A. (eds.) CRYPTO 2013, LNCS, vol. 8042, pp. 40–56. Springer (2013)

[B31] Lyubashevsky, V.: Lattice signatures without trapdoors. In: Pointcheval, D., Johansson, T. (eds.) EUROCRYPT 2012, LNCS, vol. 7237, pp. 738–755. Springer (2012)

[B32] Daniel J. Bernstein, Chitchanok Chuengsatiansup, Tanja Lange, Christine van Vredendaal: NTRU Prime. https://ntruprime.cr.yp.to/ntruprime-20160511.pdf
