# Supplementary material for: Research on blockchain smart contract technology based on resistance to quantum computing attacks
Source: PLoS One. 2024 May 23;19(5):e0302325. doi: 10.1371/journal.pone.0302325 (PMC11115269; doi:10.1371/journal.pone.0302325)
Supplement: Attachment — Submitted filename: comments.pdf [file pone.0302325.s002.pdf]

The author has proposed a new algorithm to counter quantum attacks on blockchain smart contracts. He has shown mathematical model that consider a vision of quantum computers.

It is desired to be considered to incorporate the following suggestions:

1. In 6.1 it needs to justify the reason to use give setup.
2. In 6.2, transactions are performed serially. Please mention that it will affect performance.
3. Fig. 7 should be shown with a direction sign,.
